# Comparative transcriptome analysis of the invasive weed *Mikania micrantha* with its native congeners provides insights into genetic basis underlying successful invasion

**DOI:** 10.1186/s12864-018-4784-9

**Published:** 2018-05-24

**Authors:** Wuxia Guo, Ying Liu, Wei Lun Ng, Pei-Chun Liao, Bing-Hong Huang, Weixi Li, Chunmei Li, Xianggang Shi, Yelin Huang

**Affiliations:** 1 State Key Laboratory of Biocontrol and Guangdong Provincial Key Laboratory of Plant Resources, School of Life Sciences, Sun Yat-sen University, 135 Xingang West Road, 510275 Guangzhou, Guangdong People’s Republic of China; 2 Department of Life Science, National Taiwan Normal University, Taipei, Taiwan

**Keywords:** Invasion, Eco-adaptation, Positive selection, Comparative transcriptomics, *Mikania*

## Abstract

**Background:**

*Mikania micrantha* H.B.K. (Asteraceae) is one of the world’s most invasive weeds which has been rapidly expanding in tropical Asia, including China, while its close relative *M. cordata*, the only *Mikania* species native to China, shows no harm to the local ecosystems. These two species are very similar in morphology but differ remarkably in several ecological and physiological traits, representing an ideal system for comparative analysis to investigate the genetic basis underlying invasion success. In this study, we performed RNA-sequencing on the invader *M. micrantha* and its native congener *M. cordata* in China, to unravel the genetic basis underlying the strong invasiveness of *M. micrantha*. For a more robust comparison, another non-invasive congener *M. cordifolia* was also sequenced and compared.

**Results:**

A total of 52,179, 55,835, and 52,983 unigenes were obtained for *M. micrantha*, *M. cordata*, and *M. cordifolia*, respectively. Phylogenetic analyses and divergence time dating revealed a relatively recent split between *M. micrantha* and *M. cordata*, i.e., approximately 4.81 million years ago (MYA), after their divergence with *M. cordifolia* (8.70 MYA). Gene ontology classifications, pathway assignments and differential expression analysis revealed higher representation or significant up-regulation of genes associated with photosynthesis, energy metabolism, protein modification and stress response in *M. micrantha* than in *M. cordata* or *M. cordifolia*. Analysis of accelerated evolution and positive selection also suggested the importance of these related genes and processes to the adaptability and invasiveness of *M. micrantha*. Particularly, most (77 out of 112, i.e. 68.75%) positively selected genes found in *M. micrantha* could be classified into four groups, i.e., energy acquisition and utilization (10 genes), growth and reproduction (13 genes), protection and repair (34 genes), and signal transduction and expression regulation (20 genes), which may have contributed to the high adaptability of *M. micrantha* to various new environments and the capability to occupy a wider niche, reflected in its high invasiveness.

**Conclusions:**

We characterized the transcriptomes of the invasive species *M. micrantha* and its non-invasive congeners, *M. cordata* and *M. cordifolia*. A comparison of their transcriptomes provided insights into the genetic basis of the high invasiveness of *M. micrantha*.

**Electronic supplementary material:**

The online version of this article (10.1186/s12864-018-4784-9) contains supplementary material, which is available to authorized users.

## Background

When a species is introduced into a new environment, it either does not adapt and quickly goes extinct or persists and establishes in the new environment. Species that live on may become competitive and colonize new areas and niches at high rates, eventually becoming successful invaders. Since many invasive species pose serious threat to native biodiversity and cause severe economic loss in the affected regions [1–5], biological invasion has long been recognized as a leading threat to the functioning of local ecosystems and global biodiversity [6, 7]. As transmission of biological material increases worldwide due to the development of international trade and more frequent human activity [5, 8], such crisis becomes increasingly severe. Understanding the mechanisms by which invasive plants succeed would eventually be useful for control efforts [9] and thus are of great importance and necessity. Over the past decades, extensive insights have been gained on the biology or ecology of plant invasion, which suggests several factors to be responsible for invasive success, including external environmental conditions (e.g., lack of natural enemies, increased anthropogenic disturbance, and a wide range of invasive habitats) and their intrinsic biological characteristics (e.g., the capability of rapid reproduction, broad eco-adaptability, and strong allelopathic effects) [10–15]. However, the relative lack of genomic data for invasive plants, which represent mostly non-model species, hinders research on this group of organisms at the molecular level. As the development of transcriptome sequencing technology offers a convenient and efficient means to obtain genome resources in non-model species [16–18], it provides an opportunity for comparative study of closely related invasive and non-invasive congeners, as an effective approach to identify the genetic basis and mechanisms of invasive success [9, 19, 20].

*Mikania micrantha* H.B.K. is a perennial vine that belongs to the family Asteraceae. This species is native to tropical America, and has been recognized as one of the world’s most notorious invaders [9, 21]. With its extremely fast growth and ability of both sexual and asexual reproduction, *M. micrantha* can rapidly colonize disturbed habitats, while competing with the native vegetation and retarding their growth [5, 22–24]. Due to both external (e.g., human activity) and internal (e.g., strong invasiveness) reasons, the plant is now widely distributed across tropical Asia and the Pacific Islands, causing serious economic and environmental impacts [5, 13, 21, 25–29]. Among the more than 400 *Mikania* species, most of which native to tropical America, *M. micrantha* is the only species that has spread from the New World to the Old World, and now coexists with its native congener in the Old World, *M. cordata* (Burm. f.) B. L. Robinson [30–33]. The earliest record of *M. micrantha* in the Old World could be traced back to 1884 in Hong Kong, and after then, *M. micrantha* has expanded in southern China, covering Hong Kong and the Guangdong and Taiwan provinces [33]. In contrast, *M. cordata* is the only *Mikania* species native in South China, distributed in Yunnan, Hainan, and Taiwan provinces [13, 34, 35]. Interestingly, although *M. micrantha* and *M. cordata* appear to be quite similar to each other in morphology and life style, they show considerable differences in many ecological traits, such as niche requirements, eco-adaptability, and most importantly, invasiveness. As observed in Taiwan where they co-exist, for example, while *M. micrantha* run rampant and becomes a major pest of crops and forests [36, 37], *M. cordata* grows slowly and exhibits no harm to other native species and the local ecosystems [38].

Previous studies on the invasion of *M. micrantha* were mainly performed on the species alone and/or focused on its ecological impacts and physiological traits [9, 35, 39–49]. Comparisons between *M. micrantha* and its non-invasive congeners that share morphological and life-history traits is lacking [9, 38, 46, 47]. Among the few comparative studies, one of the most compelling observations is the higher photosynthetic efficiency and capability of acclimation to light observed in *M. micrantha* compared to *M. cordata* [9, 24, 28, 47], which is considered to be at least partly associated with the invasiveness of *M. micrantha*. Studies on habitat preferences have revealed that *M. micrantha* can invade a relatively wider light niche, while *M. cordata* can only tolerate shady environments in tropical and subtropical China [9, 38, 47, 50, 51]. *M. micrantha* has also been found to be able to achieve extremely fast growth and reproduction in new environments [52], hinting that efficient response to new abiotic and biotic stresses, such as climate, soils, and pathogens [53, 54] followed by successful adaptation and range expansion, could be another remedy for its invasiveness. Yang et al. (2017) has also preliminarily associated the ecological adaptation of *M. micrantha* to different habitats with differential expression of genes involved in high light intensity stress response, protein folding, and oxidative processes by comparing native and introduced *M. micrantha* populations [49]. While Huang et al. (2012) also provided a preliminary insight of the *M. micrantha* transcriptome, constrained by the sequencing technology and bioinformatics tools developed then, the size and quality of data obtained at that time was relatively limited for a thorough study [55]. Although these studies have undoubtedly extended our understanding on the invasiveness of *M. micrantha* from the physiological and ecological perspectives (i.e., the physiological plasticity and rapid adaptive regulation), the underlying molecular mechanisms remains barely explored.

Different from intraspecific analysis on gene expression or other physiological characteristics, which has been a common method to investigate the adaptive strategies of invasive species [9, 24, 28, 47, 49, 56], genomic-level interspecific comparisons of closely related invasive and non-invasive congeners would offer further insights into the molecular mechanisms underlying those traits or the genetic basis that confer greater invasive potential to a species. The species pair of *M. micrantha* and *M. cordata* thus offers an ideal system for comparative analysis. To further exclude possible biases due to stochastic factors or phylogenetic effects from comparing only two species, the inclusion of a third, closely related, non-invasive congener in such a study would aid in the reduction of these stochastic effects to more accurately identify the genomic/transcriptomic features that is unique to the invasive species. In this study, we performed RNA-sequencing (RNA-seq) and assembled the transcriptomes of the invasive plant *M. micrantha* and its coexisting non-invasive congener *M. cordata* from China, and another allopatric non-invasive congener *M. cordifolia* [57, 58]. In order to construct a phylogenetic framework for downstream comparisons, the relationship among the three species has to be first established. For that, we conducted a phylogenetic analysis and estimated the time and level of divergence among the species. We then performed a series of detailed comparative analyses including on the differences in genomic contents and changes in gene sequences, and assessed the role of evolutionary factors (e.g., natural selection) on the adaptation and invasion potential of *M. micrantha*. The objective of this study was to use large-scale datasets to identify the genetic basis of invasiveness observed in *M. micrantha*, which would be valuable for further unraveling the molecular mechanisms of invasion success and contribute to the control efforts of the species. In particular, we explored: (1) the degree of sequence differentiation among *M. micrantha*, *M. cordata*, and *M. cordifolia*, and the divergence times of these three species; (2) the differences in gene components and expression patterns between the transcriptomes of *M. micrantha* and *M. cordata*/*M. cordifolia* and their functional roles; and (3) the evolutionary signatures of *M. micrantha* genes, i.e., the types of genes with putatively accelerated nonsynonymous divergence and genes that show signals of positive selection, and their potential correlations with the adaptability and invasiveness of *M. micrantha*.

## Results

### Transcriptome sequencing and de novo assembly

Using RNA-seq, a total of 41.26, 48.76, and 46.76 million paired-end reads were generated from the *M. micrantha*, *M. cordata*, and *M. cordifolia* leaf transcriptomes, respectively (Table 1). After trimming and removing low-quality bases and adapter-containing reads, 41.04, 48.76, and 45.53 million high-quality reads data remained for *M. micrantha*, *M. cordata*, and *M. cordifolia*, with Q20 percentages (sequencing error rate < 1%) of 97.51, 92.34 and 97.37%, respectively. Based on these clean reads, 75,179, 76,344, and 93,872 contigs were de novo assembled for *M. micrantha*, *M. cordata*, and *M. cordifolia*, respectively, which further resulted in 62,145, 56,409, and 53,330 non-redundant sequences with N50 values of 975, 1399, and 1561 bp, respectively, suggesting good integrity of the three assemblies. All sequencing reads generated in this study have been deposited in NCBI Sequence Read Archive (SRA) under the accession numbers SRX3520663- SRX3520665.

**Table 1:** Sequencing, assembly, and annotation statistics for the transcriptomes of *M. micrantha*, *M. cordata*, and *M. cordifolia*

	*M. micrantha*	*M. cordata*	*M. cordifolia*
Total raw reads (million)	41.26	48.76	46.76
Total filtered reads (million)	41.04	48.76	45.53
% G/C	43.98	45.32	48.50
Number of contigs	75,179	76,344	93,872
Number of non-redundant sequences	62,145	56,409	53,330
Number of unigenes	52,179	55,835	52,983
Maximum length of unigenes (bp)	6763	14,332	10,914
Average length of unigenes (bp)	643	843	1016
N50 of unigenes (bp)	1047	1406	1563
Number of total annotated unigenes	32,418 (62.13%)	31,944 (57.21%)	40,991 (77.37%)
Number of unigenes with NR annotations	29,934 (57.37%)	29,829 (53.42%)	39,576 (75.00%)
Number of unigenes assigned with GO terms	19,945 (38.22%)	27,561 (49.36%)	31,178 (58.85%)
Number of unigenes assigned to KEGG pathways	10,787 (20.67%)	9164 (16.41%)	23,381 (44.13%)
Number of unigenes with KOG annotations	11,127 (21.32%)	11,497 (20.59%)	29,854 (56.35%)

To ensure the reliability of our data and results, we first identified the putative origin (i.e., plant, animal, fungus, bacteria, archaea, virus and viroids, or other) of each non-redundant sequence based on its top-hit BLAST result against the NCBI non-redundant protein (NR) database. The BLAST results showed that for *M. micrantha*, *M. cordata*, and *M. cordifolia*, 39,511, 30,380, and 39,893, respectively, of the non-redundant sequences had hits in NR databases. As expected, most sequences (75.76, 98.19, and 99.21% of all matched sequences) of the three assemblies were of plant origins. A considerable amount of fungus-origin sequences was identified from the *M. micrantha* assembly (8917, 22.57%) while only some were found in the *M. cordata* (23, 0.076%) and *M. cordifolia* (24, 0.060%) assemblies (Additional file 1). To maximize the accuracy of our data analyses, we then removed the sequences with non-plant-origin. The filtered sequences were considered as non-redundant sequences derived from the three plant species (hereafter ‘unigenes’) and used in the downstream comparative analyses. As a result, the final unigene sets of *M. micrantha*, *M. cordata*, and *M. cordifolia* consisted of 52,179, 55,835, and 52,983 sequences, with similar length and GC distribution patterns (Additional file 2), and N50 values of 1047, 1406, and 1563 bp, respectively (Table 1).

To assess the per-base sequence accuracy of the three unigene sets, firstly, all usable reads were mapped to the unigenes and the coverage depth was counted for each site of each sequence. The results showed that, for *M. micrantha*, *M. cordata*, and *M. cordifolia*, 99.94, 99.24, and 98.03% sites in total were covered by at least one high-quality base (Phred quality score ≥ 30), equivalent to an accuracy of ≥99.9% at these sites; and 77.56, 61.03, and 76.04% sites were covered at least ten times by high-quality bases, equivalent to an accuracy of ≥99.99% (Additional file 3). Additionally, all usable nucleotide sequences available in the NCBI databases so far for the three species (61, 5, and 29 sequences from 20, 4, and 4 different genes, respectively) were obtained and compared with their corresponding unigenes assembled above. As a result, *M. micrantha*, *M. cordata*, and *M. cordifolia* unigenes showed 98.23–100%, 99.91–100%, and 98.34–100% identity with those from public databases, respectively (Additional files 3 and 4). Finally, for all three species, DNA fragments of 9 randomly chosen unigenes were amplified and sequenced by Sanger sequencing. As a result, all unigene sequences of the three species showed 100% identity with their respective Sanger-sequencing-derived sequences, and all interspecific single nucleotide polymorphisms identified from unigene sequences were also verified (Additional file 5). These results indicated high accuracy of most assembled unigenes at the per-base level and minor nucleotide discordance in a few sequences, which should have little effect on the following analyses and results given its small proportion and low degree of variation.

### Phylogenetic analysis and divergence time estimation

**Fig. 1:**
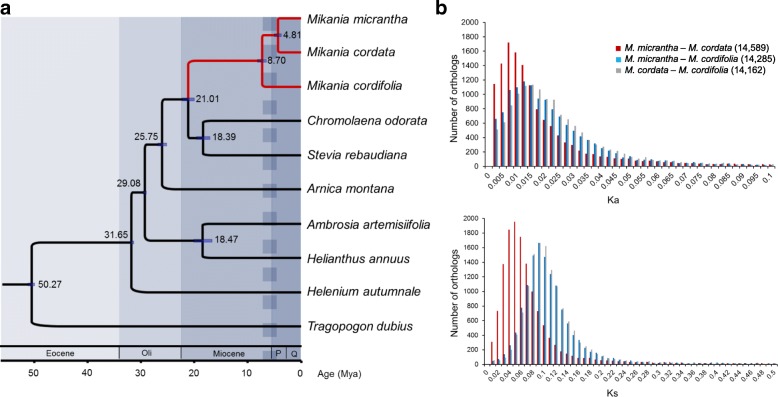
Phylogeny and divergence times of *M. micrantha*, *M. cordata*, and *M. cordifolia*. **a** Phylogeny and divergence times for the three *Mikania* species and seven other species in Asteraceae. The value and purple bar at each node indicate the estimated divergence time with a 95% credibility interval. **b** Distribution of nonsynonymous (Ka) and synonymous (Ks) substitution rates of ortholog pairs between the three *Mikania* species. The numbers in parentheses after the species name indicate the number of ortholog pairs used for plotting

To investigate the phylogenetic relationships and divergence times for *M. micrantha*, *M. cordata*, and *M. cordifolia*, a total of 456 1:1 orthologous genes were identified, based on which, a phylogenetic tree was reconstructed for the three *Mikania* species (Eupatorieae, Heliantheae alliance) and six other species from the Heliantheae alliance, including two species from Eupatorieae (*Chromolaena odorata* and *Stevia rebaudiana*), two species from the sister tribe Heliantheae (*Ambrosia artemisiifolia* and *Helianthus annuus*) and one species for each of the sister tribe Madieae (*Arnica montana*) and Helenieae (*Helenium autumnale*), using *Tragopogon dubius* (Cichorioideae) as an outgroup. As observed from the phylogenetic tree (Fig. 1a), *M. micrantha* and *M. cordata* showed a closer relationship with each other than with *M. cordifolia*. Bayesian estimation of divergence time with confident constraints further suggested that *M. cordifolia* diverged from the *Mikania* common ancestor approximately 8.70 MYA (95% credibility interval, CI = 7.5–10.0 MYA), and that *M. micrantha* and *M. cordata* split approximately 4.81 MYA (95% CI = 4.0–5.7 MYA).

For further assessment of the genetic divergence among the three *Mikania* species, their differences in sequences were also measured. Direct comparison on protein sequences of orthologous gene pairs showed an overall similarity of approximately 94.44% for *M. micrantha*-*M. cordata*, slightly higher than that for *M. micrantha*-*M. cordifolia* (93.67%) and *M. cordata*-*M. cordifolia* (93.65%) (Additional file 6). Further estimation under the nucleotide substitution model revealed that the median nonsynonymous substitution rate (Ka) was 0.0125 (Fig. 1b), indicating that about 1% of each protein sequence differs, between *M. micrantha* and *M. cordata*, which was lower than that between *M. micrantha* and *M. cordifolia* (Ka = 0.0183) or between *M. cordata* and *M. cordifolia* (Ka = 0.0195). As for the silent site divergence, the median synonymous substitution rate (Ks) was 0.0560 between *M. micrantha* and *M. cordata*, 0.0976 between *M. micrantha* and *M. cordifolia*, and 0.0980 between *M. cordata* and *M. cordifolia* (Fig. 1c). These results collectively suggested a closer relationship, i.e., smaller divergence, between *M. micrantha* and *M. cordata* than any of them with *M. cordifolia*.

### Functional annotation and GO classification

To understand the functions of the unigenes derived from the three *Mikania* species in this study, we performed similarity search on these sequences with those in public databases. In total, 32,418 (62.13%), 31,944 (57.21%), and 40,991 (77.37%) of *M. micrantha*, *M. cordata*, and *M. cordifolia* unigenes had matches in at least one database (Table 1; Additional file 7), indicating that our assemblies covered a substantial number of genes of the two species. For *M. micrantha*, *M. cordata*, and *M. cordifolia*, among the 29,934 (57.37%), 29,829 (53.42%), and 39,576 (75.00%) unigenes with matches in the NR database, 15,377 (51.37%), 24,624 (82.55%), and 33,820 (85.46%) unigenes matched sequences from their respective top ten species, respectively (Additional file 7). Besides, while half of these top species for *M. micrantha* were unsurprisingly of Asterids, most of those for *M. cordata* and *M. cordifolia* belonged to Rosids, indicating potential differences in their genetic components. Besides, the three species showed similar patterns of E-value and sequence similarity in the BLAST hit results (Additional file 7).

**Fig. 2:**
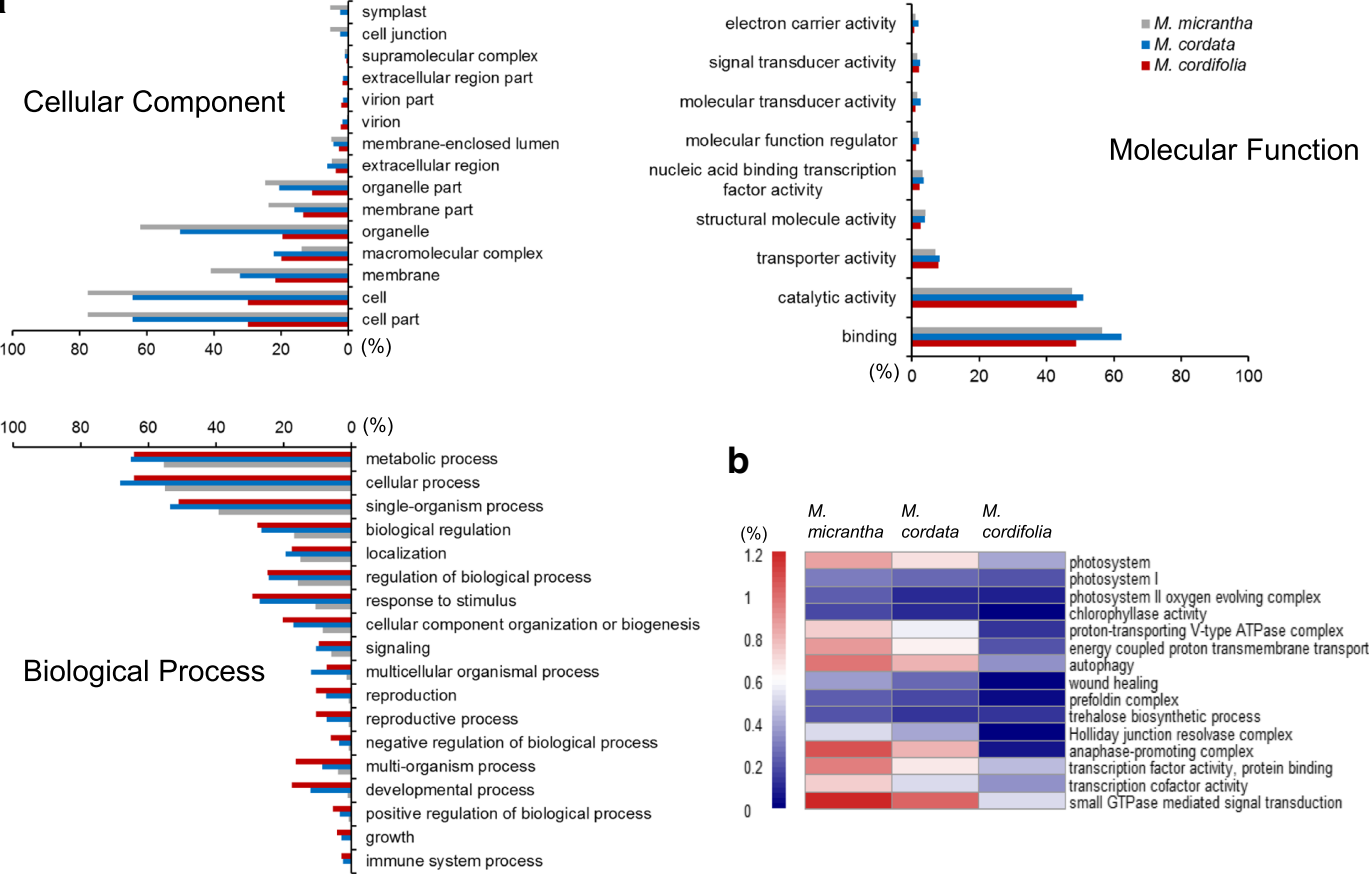
Gene ontology (GO) classification and enrichment analysis for *M. micrantha*, *M. cordata*, and *M. cordifolia*. **a** Gene distribution at GO level two. Shown are GO terms classified into tree main categories (i.e., cellular component, molecular function, and biological process) and containing 1% or more of total genes in at least one of the three species. **b** Functional categories overrepresented in *M. micrantha*. The heat map shows the representative GO types significantly enriched in *M. micrantha* compared with both *M. cordata* and *M. cordifolia* (Fisher’s exact test and FDR, *P* < 0.05). Colors represent the percentage of genes in the corresponding categories

Based on the above BLAST search results, GO annotations were then retrieved. For *M. micrantha*, *M. cordata*, and *M. cordifolia*, 19,945 (38.22%), 27,561 (49.36%), and 31,178 (58.85%) unigenes had GO terms assigned in the three main categories, including 14,388, 22,144, and 24,909 unigenes with terms from “Biological Process”, 9226, 20,092, and 26,490 from “Cellular Component”, and 17,045, 23,334, and 23,811 from “Molecular Function”, respectively. While the gene distribution patterns (at level two) were generally similar among the three transcriptomes (Fig. 2a), detailed comparisons for each functional class (at all GO levels) identified significant differences of gene components among the three *Mikania* transcriptomes (Fig. 2b; Additional file 8). In total, the enrichment analysis showed 248 and 879 *M. micrantha* GO terms significantly differing from that of *M. cordata* and *M. cordifolia*, respectively. Among which, 235 and 868 terms, respectively, were overrepresented in *M. micrantha* (Fisher’s exact test and false discovery rate (FDR), *P* < 0.05), with most of them (213 out of 235) being shared (Additional file 8). It is worth noting that many of these GO classes enriched in *M. micrantha* are functionally related to photosynthesis, gene transcription/translation, cell cycle, protein modification, and damage repair, such as those involved in “photosystem I reaction center” (GO:0009538, 27 vs. 21 and 12), “photosystem II oxygen evolving complex” (GO:0009654, 44 vs. 34 and 29), “transcription factor activity, protein binding” (GO:0000988, 192 vs. 182 and 139), “protein binding involved in protein folding” (GO:0044183, 23 vs. 17 and 7), wound healing (GO:0042060, 76 vs. 72 and 1), and “metaphase/anaphase transition of cell cycle” (GO:0044784, 21 vs. 20 and 20).

### KEGG analysis and KOG classification

To investigate the active biological pathways of the three *Mikania* species, KEGG pathway assignments and detailed statistical analysis were performed. For *M. micrantha*, *M. cordata*, and *M. cordifolia*, 10,787 (20.67%), 9164 (16.41%), and 23,381 (44.13%) unigenes were assigned 43 KEGG pathway classes comprising 349, 339, and 350 subclass pathways, respectively (Table 1). Although covering similar ranges of pathway classes, the involved genes of the three species also showed differential distributions in several pathways, especially between *M. micrantha*/*M. cordata* and *M. cordifolia* (Additional file 9). As revealed by statistical analysis for each subclass pathway, *M. micrantha* genes only differed in two subclass pathways compared with *M. cordata*, while a total of 119/110 pathways were significantly different between *M. micrantha*/*M. cordata* and *M. cordifolia* (Fisher’s exact test and FDR, *P* < 0.05). Despite the more similar gene distribution patterns between *M. micrantha* and *M. cordata* than between these two species with *M. cordifolia*, which was in accordance to their phylogenetic relationships as mentioned above, it was worth noting that *M. micrantha* pathway-involving genes showed significantly higher representation than *M. cordata* and *M. cordifolia* genes in photosynthesis (ko00195; 18.26% vs. 10.27%; Fisher’s exact test and FDR, P < 0.05), a subclass pathway involved in the energy metabolic pathway.

For more comprehensive annotation, all unigenes of the three *Mikania* species were subjected to a search against the KOG database. For *M. micrantha*, *M. cordata*, and *M. cordifolia*, 11,127 (21.32%), 11,497 (20.59%) and 29,854 (56.35%) unigenes were classified into 26 KOG categories, respectively (Table 1; Additional file 10). Similar to that in GO terms and KEGG pathways, differences of gene distributions in some KOG categories were also observed among the three species, especially between *M. micrantha*/*M. cordata* and *M. cordifolia*. For example, for both *M. micrantha* and *M. cordata*, the most represented functional category was “Posttranslational modification, protein turnover, chaperones” (2161 genes, 19.42% and 1590 genes, 13.83%), followed by “Translation, ribosomal structure and biogenesis” (1487 genes, 13.36% and 786 genes, 6.84%) and “Signal transduction mechanisms” (1336 genes, 12.01% and 1114 genes, 9.69%), while that for *M. cordifolia* was “Signal transduction mechanisms” (3958 genes, 13.26%), followed by “Posttranslational modification, protein turnover, chaperones” (3126 genes, 10.47%) and “Transcription” (2179 genes, 7.30%).

### Detection of differentially expressed genes

To investigate the patterns of gene expression in the invasive and non-invasive *Mikania* species, the RNA-seq data of *M. micrantha*, *M. cordata*, and *M. cordifolia* were separately mapped to their reference transcriptomes (i.e., unigene sets), and genes differentially expressed between *M. micrantha* and *M. cordata*/*M. cordifolia* were identified. Under a priori replicate variance value of 0.2 and an FDR cut off of 0.01 (Additional file 11), 2088 and 2344 genes exhibited differential expression in *M. micrantha*-*M. cordata* and *M. micrantha*-*M. cordifolia*, with 1337 and 1724 genes up-regulated and 751 and 620 genes down-regulated in *M. micrantha* compared with *M. cordata* and *M. cordifolia*, respectively. Among them, 650 up-regulated and 241 down-regulated genes were shared between the two comparisons (Additional file 12). After GO term assignment, these shared up-regulated genes were found to be involved in 1747 GO categories (at all levels), including “response to stress”, “response to starvation”, “defense response”, and “response to DNA damage stimulus” (Additional file 13).

### Accelerated evolution analysis and identification of positively selected genes

To examine the type of genes that showed accelerated evolution and also identify genes under positive selection pressure for the three *Mikania* species, a total of 4513 orthologs within a group of five Eupatorieae (i.e., *M. micrantha*, *M. cordata*, *M. cordifolia*, *C. odorata*, and *S. rebaudiana*) and one Madieae (i.e., *A. montana*, as an outgroup) species was identified. These genes were functionally grouped into 642 different categories according to their GO annotations and used to detect the types of genes showed accelerated evolution. To infer average rates of protein evolution for different GO categories between invasive and non-invasive *Mikania* species, both *M. micrantha*-*M. cordata* and *M. micrantha*-*M. cordifolia* ortholog pairs were compared. For those between non-invasive *Mikania* species, *M. cordata*-*M. cordifolia* ortholog pairs were compared. Preliminary analysis on Ka/Ks ratios of nonsynonymous-to-synonymous substitutions for each group of genes revealed elevated *Mikania* pairwise Ka/Ks values in diverse functional categories (top 10% fastest evolving categories; Additional file 14), among which, genes related to carbon fixation and chlorophyll biosynthetic were notably present in the fastest evolution categories between invasive and non-invasive *Mikania* (i.e., both *M. micrantha*-*M. cordifolia* and *M. micrantha*-*M. cordata*) while being absent in those between non-invasive *Mikania* (i.e., *M. cordata*-*M. cordifolia*).

**Fig. 3:**
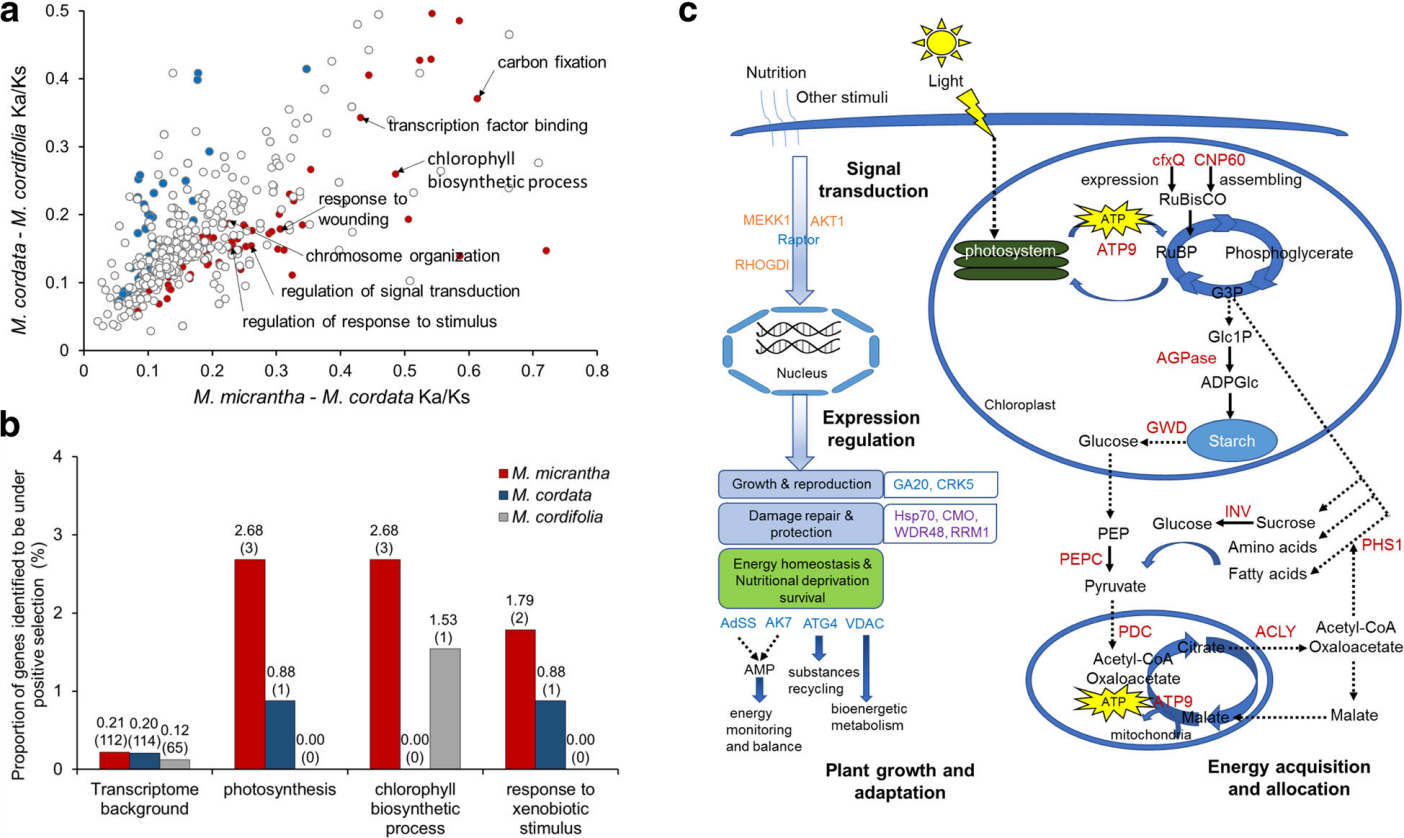
Evolutionary signals in *M. micrantha*. **a** *Mikania* pairwise Ka/Ks for each GO term. Data points represent average Ka/Ks ratios of *M. micrantha*-*M. cordata* and *M. cordata*-*M. cordifolia* pairs by GO category. GO categories with putatively accelerated (P < 0.05, Fisher’s exact test) nonsynonymous divergence in *M. micrantha*-*M. cordata* are highlighted in red while those in *M. cordata*-*M. cordifolia* are in blue. **b** Comparison of positively selected genes (PSGs) in *M. micrantha*, *M. cordata*, and *M. cordifolia*. The number of PSGs shown in different categories were obtained based on their GO classification and are given in parentheses. **c** Positively selected genes involved in energy utilization and stimuli response processes. Genes that showed evidence of positive selection in *M. micrantha* are in red, blue, purple, and orange for group I, II, III, and IV PSGs, respectively. Solid lines indicate direct relationships between enzymes and metabolites, while dashed lines indicate that more than one step is involved in the process

Further statistical comparisons showed that the average Ka/Ks in several categories, e.g., carbon fixation (0.61 vs. 0.37), chlorophyll biosynthetic process (0.49 vs. 0.26), cellular response to stress (2.77 vs. 1.66), DNA repair (11.38 vs. 5.55), chromosome organization (0.23 vs. 0.19), and transcription cofactor activity (0.52 vs. 0.43), was significantly higher in *M. micrantha*-*M. cordata* than in *M. cordata*-*M. cordifolia* (*P* < 0.05 by Fisher’s exact test; Fig. 3a; Additional file 15). Categories with similar functions (e.g., chlorophyll biosynthetic process, 0.37 vs. 0.26) were also observed when comparing *M. micrantha*-*M. cordifolia* with *M. cordata*-*M. cordifolia*, indicating that the average rate of protein evolution for these genes is faster between invasive and non-invasive than in between non-invasive *Mikania* by a significant margin. When estimating the number of genes that showed higher Ka/Ks in *M. micrantha*-*M. cordata*/*M. cordifolia* than in *M. cordata*-*M. cordifolia* or vice versa, however, it was found that in most of these categories the number of genes with higher Ka/Ks in *M. micrantha*-*M. cordata*/*M. cordifolia* were not significantly higher than the other way around (*P* > 0.05 by the binomial test), arguing that for these categories the higher average Ka/Ks in between invasive and non-invasive *Mikania* is mainly contributed by certain genes instead of the combined effects of a large fraction of all genes involved. This implication was further supported by the statistical tests on Ka/Ks distributions, which revealed that the *M. micrantha*-*M. cordata*/*M. cordifolia* distributions were not significantly different from the *M. cordata*-*M. cordifolia* distributions (*P* > 0.05 by the Wilcoxon signed-rank test).

Lineage-specific analysis revealed relatively slower evolutionary rates for most *M. micrantha* genes than those of *M. cordata* and *M. cordifolia*, as revealed by their median Ka/Ks values of all orthologous genes calculated using the free-ratio model (i.e., Ka/Ks of 0.0981, 0.1382, and 0.1275 for *M. micrantha*, *M. cordata*, and *M. cordifolia*, respectively), which can also be observed through the distributions of these Ka/Ks values that showed relatively more *M. micrantha* genes in the very low Ka/Ks range (i.e., Ka/Ks ≤ 0.05; Additional file 16). These results might suggest that compared with *M. cordata* and *M. cordifolia*, the majority of *M. micrantha* genes were less likely to be changed while some genes might be under excess evolutionary pressure (i.e., positive selection) and of special significance for the species. To provide full lists of candidate genes that have been under positive selection in the invasive *M. micrantha* and understand their functional importance, positively selected genes (PSGs) were identified along each *Mikania* branch using the improved branch-site likelihood ratio test. In total, 213, 225, and 178 PSGs were identified, with 112, 114, and 65 genes left after correcting for multiple tests (FDR, *P* < 0.05) for *M. micrantha*, *M. cordata*, and *M. cordifolia*, respectively (Fig. 3b; Additional file 17). Of the 112 *M. micrantha* PSGs, 85 genes were assigned to 857 terms of the three main GO categories, and 43 were assigned to 72 KEGG pathways. Enrichment tests for all GO hierarchies revealed that these PSGs were significantly overrepresented compared to the background level (*P* < 0.05, Fisher’s exact test and FDR) in categories including chlorophyll biosynthesis, ATPase activity, response to stimulus, protein folding, and chromosome segregation (Additional file 18). After thorough exploration on gene functions for each PSG, most *M. micrantha* PSGs (77, 68.75%) were found to be mainly involved in processes of i) energy acquisition and utilization, ii) growth and reproduction, iii) protection and repair, and iv) signal transduction and biological regulation, which might have contributed to the specific traits, e.g., wide eco-adaptability and intense invasiveness, of *M. micrantha* (Fig. 3c).

## Discussion

### Recent divergence between the invasive *M. micrantha* and the non-invasive *M. cordata*

*Mikania* belongs to Eupatorieae under the subfamily Asteroideae and family Asteraceae, one of the largest angiosperm families with over 24,000 species [59, 60]. Huang et al. (2016) performed an elaborate analysis of the phylogeny, divergence, and polyploidization of 73 Asteraceae species, providing evidences for the correlations among polyploidization, stressful environments, and new open niches after mass extinction, as well as the species radiation of Asteraceae into large subfamilies or tribes [60]. Since their study mainly focused on the large subfamilies or tribes, divergences between small groups (e.g., within genus) were barely mentioned. In this study, further estimation of the divergence times using 456 single copy orthologous genes suggested that the split of the three *Mikania* species occurred during or soon after the late Miocene (5.3–11.2 MYA), with *M. cordifolia* diverging first (8.70 MYA, 95% CI = 7.5–10.0 MYA), followed by the split of *M. micrantha* and *M. cordata* (4.81 MYA, 95% CI = 4.0–5.7 MYA) (Fig. 1). The late Miocene is an important geological epoch for the evolution of global climate [61–63]. Severe environmental changes during the late Miocene generated a greater diversity of habitats and led to a rediversification of temperate and tropical ecosystems [64], which could be responsible for the diversification of *Mikania* through adaptive speciation.

For *M. micrantha* and *M. cordata*, particularly, the differing habitat conditions (i.e., sunny vs. semi-shaded areas) derived from the late Miocene environmental changes might have driven the subsequent divergence of their ancestral populations and promoted the accumulation of genetic differences that are of benefit for adaptation to their respective habitats. Although the present-day morphological features of *M. micrantha* and *M. cordata* are still so similar that it is difficult to distinguish them with the naked eye [47], the several million years of evolution still resulted in considerable genetic differences between the two species, as observed in differences in the number of genes (52,179 vs. 55,835) and gene sequence (Ka = 0.0125 and Ks = 0.0560) obtained in this study. Given the distinct niche requirements and different invasion capabilities between *M. micrantha* and *M. cordata*, the detected genetic divergence could be the underlying cause for the higher eco-adaptability of *M. micrantha*, giving it the potential to invade various habitats (e.g., wider light niche).

### Overrepresented functional categories may represent unique molecular characteristics associated with the invasiveness of *M. micrantha*

The successful invasion of a plant species comprises firstly the adaptation to and colonization of a new habitat, followed by the rapid range expansion in introduced region. These are greatly correlated with biological processes like acclimation to important environmental factors, response to various environmental stimuli, together with fast growth and reproduction. These complex processes usually involve multiple genes underlying the relevant biological characteristics across the transcriptome, thus a comparison of transcriptomes between the invasive species and its non-invasive congener could unravel specific genetic components that might have contributed to its successful invasion. In this study, detailed comparison of GO classifications between transcriptomes of *M. micrantha* and its indigenous sister species *M. cordata* revealed noteworthy differences between these two species. *M. micrantha* was found to harbor far more overrepresented GO categories than *M. cordata* (235 vs. 13). Notably, most of these *M. micrantha*-enriched functional categories (213 out of 235, 90.64%) also had higher representation in *M. micrantha* when compared to *M. cordifolia* (Fisher’s exact test and FDR, *P* < 0.05), and several of them were found to be involved in photosynthesis, energy metabolism, wound healing, protein modification, asexual reproduction, and biological regulation process (Additional file 8), hinting at the special significance of these functions to the physiology of the invasive *M. micrantha*. Moreover, comparison of genes in each KEGG pathway between *M. micrantha* and *M. cordata* identified an enrichment of *M. micrantha* genes involved in the photosynthesis pathway (ko00195, a subclass of energy metabolism) (Fisher’s exact and FDR, *P* < 0.05), which was, again, identified for *M. micrantha* in its comparison to *M. cordifolia* (Additional file 9). The marked differences in gene contents of these specific functions between *M. micrantha* and both of its non-invasive congers may be at least partly responsible for their differences in eco-adaptability and invasiveness.

Besides gene contents, we also tested if the differential invasiveness between species were reflected in the patterns of their gene expression. As our study primarily emphasized on the comparative transcriptomics and evolutionary analysis, the experimental design was not well suited for rigorous detection of differentially expressed genes. However, procedures implemented in EdgeR [65] allow us to gain some preliminary observations under such situation by setting a priori for the replicate variance. Thus, we used a conservative approach (i.e., assuming a high error variance) to detect genes differentially expressed between the invasive and non-invasive *Mikania* species. After trying a series of variance values, 0.2 was as the final setting. We found that the number of significantly up-regulated genes (1337 and 1724) was much larger than that of down-regulated (751 and 620) in *M. micrantha* compared with *M. cordata* or *M. cordifolia* (Additional files 11). This trend was still obvious when considering the genes jointly up-regulated (650 genes) or down- regulated (241 genes) in *M. micrantha* compared with both *M. cordata* and *M. cordifolia*, suggesting that these up-regulated genes might have important functions to the invasive characteristics of *M. micrantha* (Additional file 12). Statistical tests (FDR < 0.05) indicated that functional categories such as chlorophyllase activity, response to stress, response to nutrient levels, protein processing and DNA repair were significantly overrepresented in this set of genes Additional file 13). Notably, although a conservative method was adopted, the differentially expressed genes detected here should still be treated with caution because of the lack of biological replicates. Combined with the gene contents patterns discussed above, it is reasonable to speculate that the higher representation of these genes might be beneficial for improving the photosynthetic rate, energy and resources supply, damage repair capability, and other cellular regulation efficiency of *M. micrantha*.

### Gene classes showing accelerated evolution between *M. micrantha* and congeners may be important for invasiveness

Besides the differences in genetic components as discussed above, characterization of the patterns of sequence divergence across the genomes could further help to clarify the evolutionary processes that gave rise to the distinct features between species, and even the genetic basis and mechanisms of adaptive evolution and speciation [66–68]. Rapid evolutionary change, i.e., accelerated evolution, has been shown to be a molecular correlate of several biological phenomenon such as phenotypic evolution, population divergence, and adaptive evolution [69–72]. In this study, pairwise analysis of nonsynonymous-to-synonymous substitutions for different GO categories revealed that the average evolutionary rates were significantly higher in *M. micrantha*-*M. cordata* than in *M. cordata*-*M. cordifolia* (*P* < 0.05 by Fisher’s exact test) for genes in carbon fixation, chlorophyll biosynthesis, cellular response to stress, DNA repair, and transcription regulation (Additional file 15). As several of these similar functional categories were also observed for *M. micrantha*-*M. cordifolia* in comparison to *M. cordata*-*M. cordifolia*, these results jointly indicated that these gene classes generally evolved faster between the invasive and non-invasive, than between the two non-invasive, *Mikania* species. The corresponding impacts on the related biological processes, especially photosynthesis and stress response, may then have special significance to the invasiveness of *M. micrantha*. This makes sense considering that, in contrast to their indigenous congeners, invasive plants usually face new living conditions very different from their original habitats, and successful establishment there may require efficient response to various accompanying environmental stresses, as well as more efficient energy production for rapid growth and reproduction. Another notable finding was that the higher average evolutionary rate of these functional classes was likely to be contributed by a small fraction of genes involved instead of the majority of these genes, as revealed by statistical tests on the inequality in the number of genes with higher Ka/Ks (*P* > 0.05, binomial test) and the distinction of Ka/Ks distribution between species pairs. This could be plausible considering the relatively recent divergence of these *Mikania* species, especially between *M. micrantha* and *M. cordata*.

### Positive selection of key genes in *M. micrantha* could have contributed to its invasiveness

As lineage-specific Ka/Ks analysis showed a slower overall rate of protein evolution in *M. micrantha* (median Ka/Ks = 0.0981) than those in *M. cordata* and *M. cordifolia* (median Ka/Ks = 0.1382 and 0.1275, respectively), these results, combined with the pairwise analysis discussed above, suggested that pronounced molecular evolutionary changes may preferentially imprint on certain genes in *M. micrantha* while the majority of genes are less likely to be changed. Since genes performing basic cellular functions conserved across different species should have evolved predominantly under constraint [70], genes under evolutionary pressures (i.e., positive selection) may be important for the specific lifestyle of a species or the adaptive traits crucial for the species’ survival and spread in stressful environments [68, 71, 72]. Therefore, having a full picture of the positively selected genes for the invasive *M. micrantha* would be very helpful for the better understanding of the molecular correlates for its strong invasiveness. To this end, signatures of positive selection were tested for all orthologous genes along each *Mikania* branch using the improved branch-site model [73]. Of the 213 genes that exhibited significant heterogeneity in Ka/Ks along the protein sequences with one or more site classes possessing Ka/Ks > 1 (Likelihood ratio test, *P* < 0.05), 112 of them showed a significant signature of positive selection after correcting for multiple tests (FDR, P < 0.05) (Additional file 17). Among which, 77 PSGs were found to be heavily relevant to the wide eco-adaptability and intense invasiveness of *M. micrantha* and could be further classified into four groups, with 10 genes in energy acquisition and utilization (group I), 13 genes in growth and reproduction (group II), 34 genes in protection and repair (group III), and 20 genes in signal transduction and biological regulation (group IV) (Fig. 3; Additional file 17), as discussed in detail below.

As an energy source and regulatory signal throughout the plant life cycle, light is among the most important environmental factors affecting the optimal growth and development of plants, especially vines [74–76]. The capability of light capture and utilization is therefore of particular relevance to the establishment, competition, and expansion of invasive plants [77]. For the invasive vine *M. micrantha*, physiological and biochemical studies have demonstrated its greater photosynthesis capability than *M. cordata* as well as several other associated species [9, 47, 78]. The underlying genetic basis, however, has never been investigated at the molecular level, until now. In this study, 10 genes under positive selection in *M. micrantha* were found to be associated with energy acquisition and utilization (i.e., photosynthesis and photorespiration) (Fig. 3; Additional file 17). Particularly, GF_1026 encodes the cfxQ (carbon fixation Q) protein homolog, known to be essential for the expression of RuBisCO (ribulose 1,5-bisphosphate carboxylase) [79]; and GF_2511 encodes the RuBisCO large subunit-binding protein subunit alpha (CNP60), which is necessary for the assembly of the RuBisCO subunits into an integrated enzyme and also functions in the folding and protection of proteins as a chloroplast chaperone protein [80, 81]. Since RuBisCO is well known as a key enzyme for carbon fixation during photosynthesis while also participating in photorespiration, these genes could be vital for the photosynthetic efficiency of plants. Besides, while GF_1627 encodes a rate-limiting enzyme of starch synthesis (glucose-1-phosphate adenylyltransferase, AGPase) [82], GF_2051 encodes an enzyme (glucan water dikinase, GWD) acting on the initial event of starch degradation [83]. Since starch is the primary energy reserve in higher plants, these genes are vital for the metabolism and growth of plants. Collectively, the positively selected group I genes may improve the net photosynthetic rate of *M. micrantha*, allowing it to acclimatize to high light environments (and thus wider light niche), which in turn provides ample carbon for growth and reproduction [9].

Another most representative characteristic of *M. micrantha* is its extremely fast growth and strong reproduction [84]. Unlike its indigenous sister species *M. cordata*, *M. micrantha* grows very fast, e.g., the stem tip can elongate up to 20 cm a day under suitable conditions in summer [37], and has strong capability of asexual (e.g., can take root anywhere in the stem node) [5, 22] and sexual reproduction (e.g., large amount of seeds that spread and germinate easily) [28]. Group II PSGs in *M. micrantha* is comprised of genes involved mainly in cell growth, shoot/root development, seed germination, energy homeostasis (e.g., during nutrient deprivation), and other hormone-related processes that regulate plant growth and development (Fig. 3; Additional file 17). For example, GF_982 encodes cysteine protease ATG4 that is required for autophagy, a strategy that eukaryotic cells use to survive nutritional deprivation through degradation and digestion of non-essential cytoplasmic materials for reuse in essential biosynthetic processes [85]. The product of another gene GF_2509 (regulatory associated protein of mTOR, raptor) is also involved in the signaling pathway that regulates cell growth in response to nutrients and growth factors; this gene has been further found to function in the controls of seed morphology, viability, and germination potential [86, 87]. Besides, GF_4172 encodes a phosphotransferase enzyme (adenylate kinase 7, AK7) that plays an important role in cellular energy homeostasis [88] and GF_3237 encodes the voltage-dependent anion channel (VDAC) protein which plays a role in mitochondrial physiology and bioenergetics metabolism [89]. Positive selection of these genes could be beneficial for improving the sensitivity and response of *M. micrantha* to nutrient levels and energy sufficiency, and contribute to its survival even in poor environments. In addition, GF_1791 encodes the D14 homolog that participates in inhibition of shoot branching, which is one of the critical determinates of aerial plant architecture [90], and GF_1505 (CDPK-related kinase 5, CRK5) is required for primary root elongation and root gravitropic response, the inactivation of which causes a root gravitropic defect and stimulates lateral root formation [91]. Positively selection of these genes might be at least partly responsible for the high reproductive allocation and phonotypic plasticity (e.g., sufficient regulation of different reproductive strategies in different living conditions) of *M. micrantha* that confers it the ability to rapidly expand in its range.

Undeniably, the capability of efficient response by an invading species when faced with new environmental stresses determines its survival in new habitats. For *M. micrantha*, successful invasion worldwide would hardly have realized without sufficient cellular protection strategies in response to various biotic and abiotic stresses. Most of the group III PSGs identified in *M. micrantha* were found to be associated with the protection and damage repair in cell, including genes involved in cell cycle control, DNA replication and repair, and the proper translation, folding, and degradation of proteins (Fig. 3; Additional file 17). GF_2718, especially, encodes the heat shock 70 kDa protein (Hsp70), which functions in facilitating the folding of nascent and denatured proteins and has been widely known to be essential for the cell to survive environmental stresses [92]. Positive selection of these genes may be essential for the accurate maintenance of genomic materials in cell division during the plant’s rapid growth, as well as the protection of cellular macromolecules to ensure normal functionality even in stressful conditions. *M. micrantha* has demonstrated the capability of dispersing in saline soils by seed and vegetative propagation and has invaded the coastal saline habitat of Guangdong and Hong Kong areas in China [5]. The two PSGs, GF_896 (stress response protein NST1-like) and GF_1847 (choline monooxygenase, CMO), might have at least partly contributed to the salt tolerance of *M. micrantha*, for NST1 may act as a negative regulator of salt tolerance, while CMO catalyzes the committing step in the synthesis of glycine betaine, a well-known osmoprotectant accumulated by many plants in response to salinity and drought [93]. Besides the functional genes discussed above, group IV PSGs mainly functions upstream of biological processes and pathways, e.g., transcription regulation and signal transduction, which may be important for the sensitivity to environmental stimuli and promote efficient downstream processes in response to these stimuli.

## Conclusion

Large-scale molecular-level comparison between the invasive *M. micrantha* and its non-invasive congeners *M. cordata* and *M. cordifolia* is informative in understanding the molecular basis of plant invasion. In this study, we generated transcriptome data for the three *Mikania* species and examined the genetic basis underlying the invasiveness of *M. micrantha*. Divergence time analysis suggested that the drastic environmental changes and the accompanied habitat diversification during the late Miocene epoch may be responsible for the diversification of *Mikania*. As revealed in this study, despite the broad similarities between *M. micrantha* and *M. cordata*/*M. cordifolia* in general patterns of gene distribution and sequence divergence, the several million years of evolution did result in remarkable differences between the invasive and non-invasive species in gene content, gene expression pattern, and gene evolutionary rate of some specific functional categories, which may have resulted in the higher eco-adaptability and invasiveness of *M. micrantha*. Moreover, evolutionary analysis suggested that positive selection has also played an important role in the evolution of *M. micrantha*’s capability of adaptation to various habitats and thus promote its invasion. This study primarily emphasized on comparative transcriptomics and evolutionary analysis, and since only one individual was selected to represent each species, inclusion of more biological replicates and more rigorous studies on differential gene expression will provide extra insight into the molecular characteristics of plant invasion. Besides, although the maturing next-generation sequencing technologies and data processing procedures provide guarantee for the quality of derived sequences, a degree of base uncertainty may still occur in some sequences. We therefore recommend examination of per-base accuracy to be performed when necessary, and their potential effects on downstream analysis, even if minimal, should be noted for better guidance of future studies. Nevertheless, findings through this study advances the current understanding of the divergence of *Mikania* species and the genetic basis of *M. micrantha*’s invasion success, which will contribute to better control and prevention efforts.

## Methods

### RNA extraction and transcriptome sequencing

Two seedlings, each representing *M. micrantha* and *M. cordata*, were collected from Taipei, Taiwan, China (24° 50′, 121° 32′). *M. cordifolia* seeds from Hillsborough Co. Florida, USA, were sown in a greenhouse, and one resulting seedling was used for RNA extraction experiments. The taxonomic identification of the plant material was undertaken by Dr. Ying Liu in Sun Yat-sen University. Voucher specimens (*Y. liu 16,283, Y. Liu 16,252*, and *Y. Liu 17,384* for the *M. micrantha*, *M. cordata* and *M. cordifolia* samples, respectively) were deposited at the herbarium of Sun Yat-sen University (SYS). The seedlings were grown under the same condition for two months before their leaves were used for RNA extraction. One individual was sampled for each species, and total RNA was extracted separately from each individual using an improved CTAB method [94] immediately after harvesting. RNA integrity was then checked through 1.0% agarose gel electrophoresis and on an Agilent 2100 Bioanalyzer (Agilent Technologies, CA, USA). The qualified RNA samples were then subjected to library construction following manufacturer’s protocol (Illumina Inc. San Diego, CA, USA). A cDNA library was constructed for each sample and paired-end sequencing was performed on an Illumina HiSeq platform.

### Data processing and de novo assembly

For each library, raw sequencing reads were first examined using FastQC [95] for quality control. To minimize sequencing errors, reads that were contaminated with adapter sequences, contained N bases accounting for > 10% of the total read length, or had low-quality (Phred value ≤5) bases > 50% of the total read length, were excluded from further analysis using in-house Perl scripts. Remaining read pairs were regarded as high-quality reads, and were de novo assembled into contigs using Trinity [96] with default parameters. The sequencing reads data has been deposited in the NCBI Sequence Read Archive (SRA) with the accession number SRX3520663- SRX3520665.

After removing contigs with lengths < 200 bp, the longest sequence of each locus was selected to represent each gene, and the resulted sequence set was regarded as the non-redundant set of transcripts. To further improve reliability, the putative origin (i.e., plant, animal, fungus, bacteria, archaea, virus and viroids, or other) of each non-redundant sequence was inferred by homology search against the NCBI non-redundant protein (NR) database (BLASTX, 1e^− 6^). Those with top-hits against sequences from non-plant organisms were excluded from downstream comparative, phylogenetic, and evolutionary analyses; while the remaining sequences were regarded as unigenes derived from the plant (‘unigenes’ hereinafter).

For all unigenes, three strategies were adopted to assess their accuracy at the per-base level. Firstly, for each species, all clean reads were mapped to the reference sequences (unigenes) using BWA [97], and the coverage depth of high quality bases (Phred quality score ≥ 30) at each site of each sequence was counted using SAMtools [98] and in-house Perl scripts. Secondly, all available nucleotide sequences of the three species in the NCBI databases were identified by keyword search; after removing chloroplast genome, microsatellite, and other unrelated sequences, the remaining sequences were downloaded and compared with their corresponding assembled unigenes using Blat [99], and their identities were obtained by examining the match or mismatch at each site. Finally, primer pairs were designed from nine randomly chosen unigenes for all three species; after DNA amplification and Sanger sequencing, these sequences were compared with their corresponding assembled unigenes, and the concordance levels were obtained. It should be noted that if a Sanger-sequencing-derived sequence contains intron region, this sequence will not be completely align to its corresponding unigene and thus the “amplified length” and “match length” will be different (Additional file 5).

### Functional annotation and enrichment analysis

To annotate the assembled unigenes, each unigene sequence was first aligned against the NR database using BLASTX with an E-value cutoff of 1e^− 6^. Gene Ontology (GO) terms were then assigned based on the top hits following the BLAST2GO [100] pipeline. Attribution of metabolic pathways and Enzyme Commission (EC) numbers was performed by mapping to the Kyoto Encyclopedia of Genes and Genomes (KEGG) database. Clustering of orthologous groups was performed by BLASTP alignments on the Eukaryotic Orthologous Group (KOG) database. Mapping of annotations to the NCBI non-redundant nucleotide (NT) database and the SwissProt database were also performed for more comprehensive annotation of assembled unigenes. To compare the genomic contents between species, GO enrichment analysis was conducted using GOBU [101]. Fisher’s exact test was used to test significance of difference between species in each KEGG pathway. Multiple comparisons were corrected using the false discovery rate (FDR) control method.

### Differential gene expression analysis

For *M. micrantha*, *M. cordata*, and *M. cordifolia*, the reads were mapped to their respective unigene sets using BOWTIE2 [102]. Only read pairs that mapped uniquely to a single locus of the reference sequences and had mapping quality larger than 20 were included in further analysis. Based on the mapping results, the number of aligned reads was counted for each sequence using HTSeq [103]. After excluding genes with CPM (count per million) less than 10 in any of the three species, differentially expressed genes were detected for the *M. micrantha*-*M. cordata* and *M. micrantha*-*M. cordifolia* species pairs using the EdgeR package [65], which treat count data with negative binomial models and provide a number of tests to detect differential expression. Since there were no biological replicates in the current data set, we set a priori value for biological replicate dispersion and used the exact test in the “classic” framework, as suggested by the EdgeR developer. After trying a series of different values, the most conservative one (i.e., 0.2) was selected to look for differentially expressed genes in this study. The GO enrichment analysis for differentially expressed genes were performed using the Fisher’s exact test in GOBU, and the resulting *P*-values were corrected for multiple testing using the FDR method.

### Sequence variation of *M. micrantha*, *M. cordata*, and *M. cordifolia* orthologs

For each species, the open reading frame and protein sequence of each unigene were obtained based on their BLASTX results against the NR database. To evaluate the sequence divergence among the three *Mikania* species, putative orthologs of each species pair were first retrieved based on the bidirectional best hits of their proteome sequences using BLASTP. For each ortholog pair, protein sequences were aligned using ClustalW2 [104] with default parameters and then back-translated to alignments of corresponding codon sequences using PAL2NAL [105]. The synonymous (Ks) and nonsynonymous (Ka) substitution rates of the ortholog pairs were estimated with the pairwise likelihood method in PAML [73].

### Identification of single-copy ortholog groups and estimation of divergence time

To reconstruct a phylogeny and estimate the times that the three *Mikania* have diverged, transcriptome data of six other species from the Heliantheae alliance (i.e., *Chromolaena odorata*, *Stevia rebaudiana*, *Ambrosia artemisiifolia*, *Helianthus annuus*, *Arnica montana*, and *Helenium autumnale*) and one species from Cichorioideae (i.e., *Tragopogon dubius*, as an outgroup) were downloaded from the NCBI database and used in the phylogeny reconstruction and divergence time estimation, referring to the work by Huang et al. (2016) [60]. Protein sequences of the ten species (i.e., *M. micrantha*, *M. cordata*, *M. cordifolia*, *C. odorata*, *S. rebaudiana*, *A. artemisiifolia*, *H. annuus*, *A. montana*, *H. autumnale*, and *T. dubius*) were combined to perform an all-against-all comparison using BLASTP with E-value cutoff of 1e^− 10^. All similar sequences were subsequently processed and clustered into gene families using OrthoMCL [106]. Putative single-copy ortholog groups among the ten species were then retrieved from the clustering results using in-house Perl scripts.

For each of the 456 ortholog groups, multiple alignments of protein sequences were performed using ClustalW2, and the corresponding coding-sequence alignments were obtained accordingly using PAL2NAL. For each species, all coding sequences from the alignments were concatenated to one supergene, based on which, the phylogenetic relationship of these ten species were reconstructed using the maximum likelihood method in PhyML [107] and a best-fit substitution model suggested by JModeltest2 [108]. Based on the single-copy ortholog groups identified and the phylogenetic tree reconstructed, the divergence times were estimated using the mcmctree program in PAML. For prior settings in age estimation, the root constraint of the ten species (i.e., the divergence between Asteroideae and Cichorioideae) was set to 49.76–50.97 MYA, the divergence of Helenieae (*H. autumnale*) from other Heliantheae alliance species was set to 30.7–31.78 MYA, and the divergence of Heliantheae was set to 28.92–29.89 MYA as suggested by the estimation of Huang et al. (2016) [60].

### Evolutionary analyses

To examine the type of genes that showed accelerated evolution among the *Mikania* species and also identify genes under positive selection, orthologous genes were identified using the bidirectional-best-hits method for the six species (i.e., *M. micrantha*, *M. cordata*, *M. cordifolia*, *C. odorata*, *S. rebaudiana*, and *A. montana*). The pairwise likelihood method in PAML was employed to calculate evolutionary parameters for each ortholog of the three *Mikania* species, including the total numbers of nonsynonymous (A) and synonymous (S) substitutions, Ka, Ks, and their ratio (i.e., Ka/Ks). After assigning GO annotations to these orthologs, the average Ka, Ks, and Ka/Ks values for each GO categories were obtained. To evaluate the statistical significance that the evolutionary rates of a group of genes differ between two species pairs (e.g., *M. micrantha*-*M. cordata* vs. *M. cordata*-*M. cordifolia*), a 2 × 2 contingency table was built, with the four entries being the total A and S values in either of the two species pairs. Fisher’s exact test was then applied to the table to test statistical significance that evolutionary rates differed between the two species pairs [70]. To evaluate the significance of the inequality in number of genes with higher Ka/Ks in one species pair versus those in the other species pair, the two-tailed binomial test was used. To assess the significance that the two sets of Ka/Ks values had distinct distributions, the nonparametric Wilcoxon signed-rank test was used. The statistic works were accomplished using custom perl scripts and R packages (for binomial test and Wilcoxon test) [109].

For lineage-specific analysis, values of Ka, Ks, and Ka/Ks were estimated for each ortholog of the three *Mikania* species using the free-ratio model implemented in the codeml module of PAML, and median values were selected to represent the lineage-specific values since the median is more robust and less influenced by outliers than the mean. To detect candidate genes that have undergone positive selection in *M. micrantha*, the optimized branch-site model implemented in the codeml module of PAML was used, with *M. micrantha* as foreground branch and all other branches in the tree as background branches. A likelihood ratio test (LRT) was performed to assess the difference between the results of null and alternative models, and the LRT *P*-values were further tested using the FDR method with a conservative criterion of 0.05 to correct for multiple comparisons. GO categories with significantly higher representation of PSGs than background levels were detected using the Fisher’s exact test in GOBU. The abundance of each PSG was measured with the normalizing statistic FPKM (fragments mapped per kilobase of exon per million reads mapped) calculated using RSEM [110].

## Additional files

Additional file 1: Top-hit species categories of the non-redundant sequences of *M. micrantha*, *M. cordata*, and *M. cordifolia* assemblies. (PDF 18 kb)

Additional file 2: Length (a) and GC (b) distributions of the assembled unigenes of *M. micrantha*, *M. cordata*, and *M. cordifolia*. (PDF 202 kb)

Additional file 3: Assessment of per-base sequence accuracy of *M. micrantha*, *M. cordata*, and *M. cordifolia* unigenes**.** (**a**) Mapping depth distribution of assembled unigene sequences. Histogram shows the percentages of bases with certain ranges of coverage depth. (**b**) Identity distribution between assembled unigenes and their corresponding sequences from public databases. (PDF 132 kb)

Additional file 4: Downloaded nucleotide sequences from public databases and their alignment statistics. (PDF 117 kb)

Additional file 5: Sanger sequencing-derived sequences and their alignment statistics. (PDF 179 kb)

Additional file 6: Sequence comparison of *M. micrantha*, *M. cordata*, and *M. cordifolia* ortholog pairs. (PDF 35 kb)

Additional file 7: Similarity search and annotation of *M. micrantha*, *M. cordata*, and *M. cordifolia* unigenes**.** (**a**) Histogram plot of the data distribution based on search against multiple public databases. (**b**/**c**/**d**) Species/E-value/similarity distributions of top hits for the unigenes based on BLAST search against NCBI non-redundant protein (NR) database. (PDF 414 kb)

Additional file 8: GO terms significantly enriched in *M. micrantha* compared with both *M. cordata* and *M. cordifolia*. Significance was tested using Fisher’s exact test and control of false discovery rate (FDR) with *P*-value cutoff of 0.05. (XLSX 37 kb)

Additional file 9: KEGG pathways with genes significantly differed between two species**.** (**a**) *M. micrantha*-*M. cordata*. (**b**) *M. micrantha*-*M. cordifolia*. (**c**) *M. cordata*-*M. cordifolia*. Significance was tested using Fisher’s exact test and control of false discovery rate (FDR) with P-value cutoff of 0.05. (XLSX 31 kb)

Additional file 10: KOG classification for *M. micrantha*, *M. cordata*, and *M. cordifolia* unigenes. A total of 11,127, 11,497, and 29,854 unigenes, respectively, were grouped into 26 clusters of ortholog group terms. (PDF 74 kb)

Additional file 11: Number of differentially expressed genes (DEGs) under different dispersion values. (PDF 49 kb)

Additional file 12: List of genes that were up- and down-regulated in *M. micrantha* compared with both *M. cordata* and *M. cordifolia*. (XLSX 246 kb)

Additional file 13: GO categories over-represented in the list of up-regulated genes in *M. micrantha*. (XLSX 46 kb)

Additional file 14: Top 10% GO categories displaying the highest Ka/Ks ratios between two species. (**a**) *M. micrantha*-*M. cordata*. (**b**) *M. micrantha*-*M. cordifolia*. (**c**) *M. cordata*-*M. cordifolia*. (XLSX 19 kb)

Additional file 15: Identification of GO categories with accelerated evolution between invasive and non-invasive *Mikania*. (**a**) *M. micrantha*-*M. cordata* vs. *M. cordata*-*M. cordifolia*. (**b**) *M. micrantha*-*M. cordifolia* vs. *M. cordata*-*M. cordifolia*. (XLSX 36 kb)

Additional file 16: Distribution of branch-specific Ka/Ks for *M. micrantha*, *M. cordata*, and *M. cordifolia*. (PDF 41 kb)

Additional file 17: Positively selected genes identified in (**a**) *M. micrantha*, (**b**) *M. cordata*, and (**c**) *M. cordifolia*. (XLSX 39 kb)

Additional file 18: Functional categories overrepresented among genes under positive selection in *M. micrantha*. Significance was tested using Fisher’s exact test and control of false discovery rate (FDR) with P-value cutoff of 0.05. (XLSX 22 kb)

## Abbreviations

CI: Credibility interval
CPM: Count per million
DEG: Differentially expressed gene
EC: Enzyme commission
FDR: False discovery rate
FPKM: Fragments per kilobase of exon region in a given gene per million mapped fragments
GO: Gene ontology
Ka: Nonsynonymous substitution rate
Ka/Ks: Ratio of nonsynonymous to synonymous substitution rates
KEGG: Kyoto Encyclopedia of Genes and Genomes
KOG: Eukaryotic Orthologous Group
Ks: Synonymous substation rate
LRT: Likelihood ratio test
MYA: Million years ago
NR: NCBI non-redundant protein
NT: NCBI non-redundant nucleotide
PSG: Positively selected gene
RNA-seq: RNA-sequencing
