# Supplementary figures and images for: Comparative transcriptome analysis of the invasive weed Mikania micrantha with its native congeners provides insights into genetic basis underlying successful invasion
Source: BMC Genomics. 2018 May 24;19:392. doi: 10.1186/s12864-018-4784-9 (PMC5968712; doi:10.1186/s12864-018-4784-9)

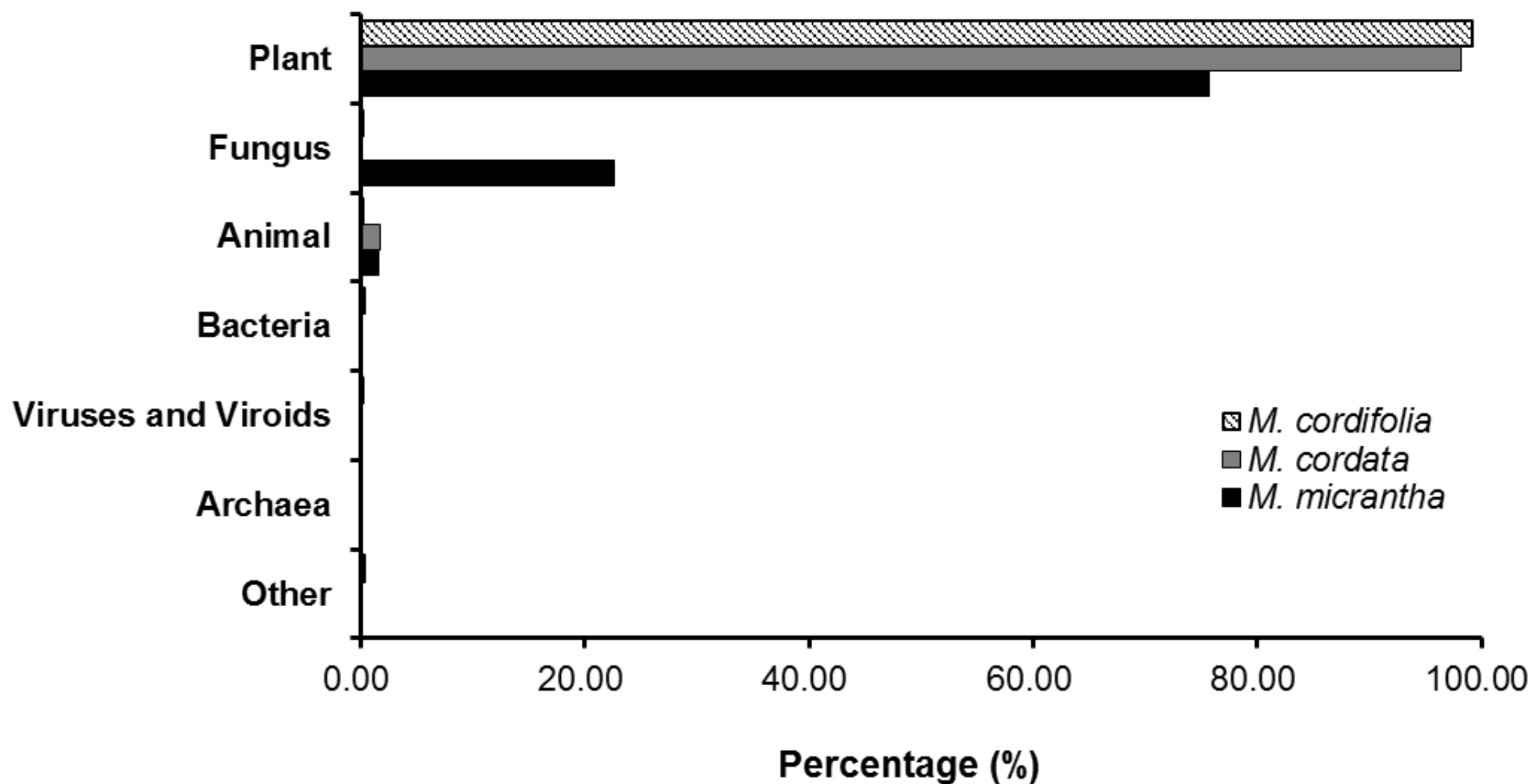

Supplement: Supplementary file 1 — Top-hit species categories of the non-redundant sequences of M. micrantha, M. cordata, and M. cordifolia assemblies. (PDF 18 kb) [file 12864_2018_4784_MOESM1_ESM.pdf]

a

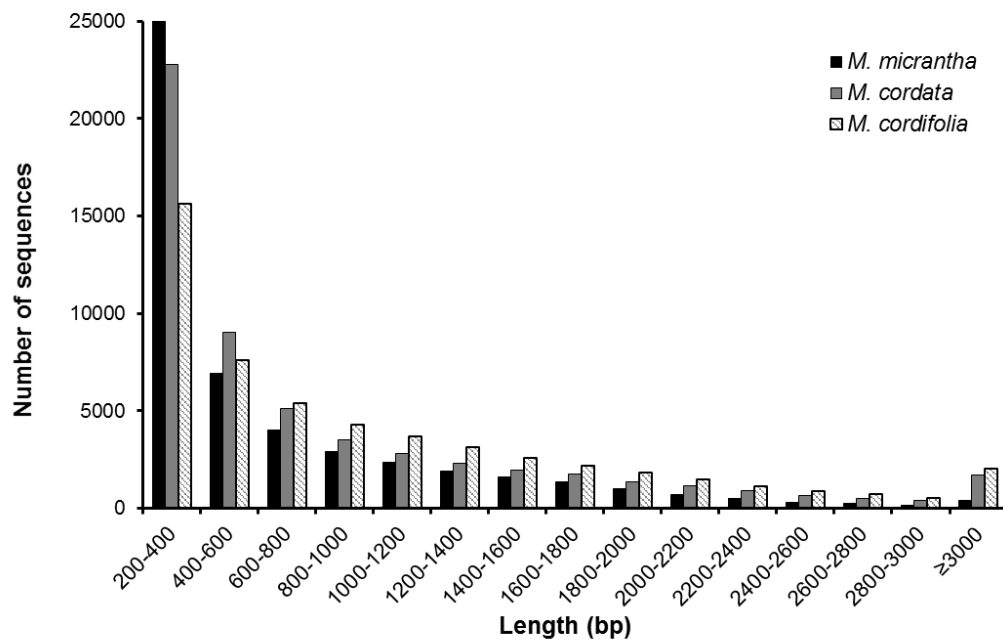

b

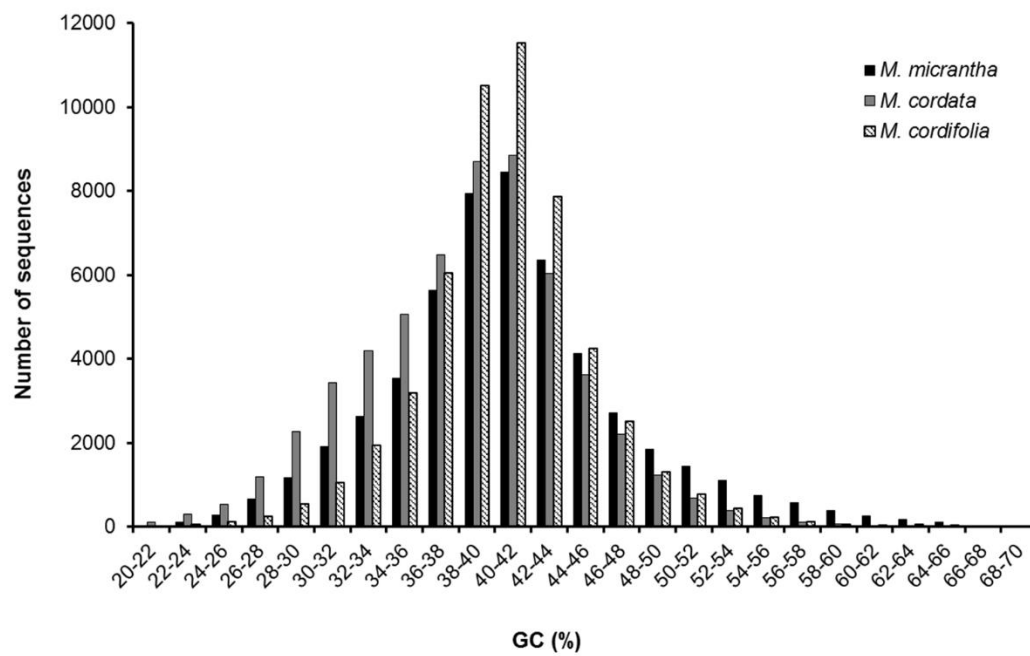

Supplement: Supplementary file 2 — Length (a) and GC (b) distributions of the assembled unigenes of M. micrantha, M. cordata, and M. cordifolia. (PDF 202 kb) [file 12864_2018_4784_MOESM2_ESM.pdf]

a

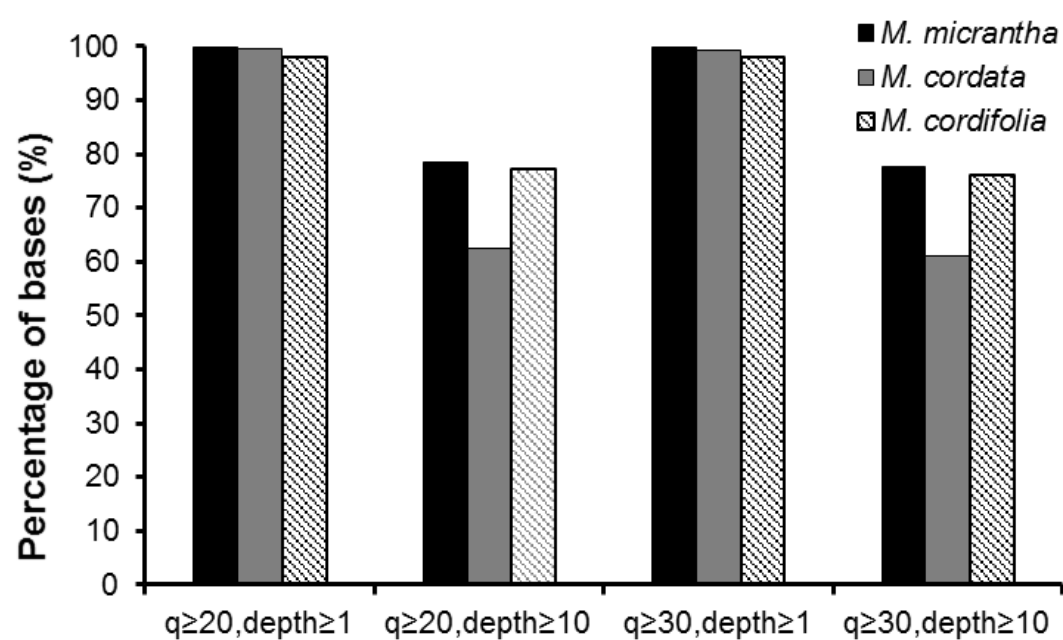

b

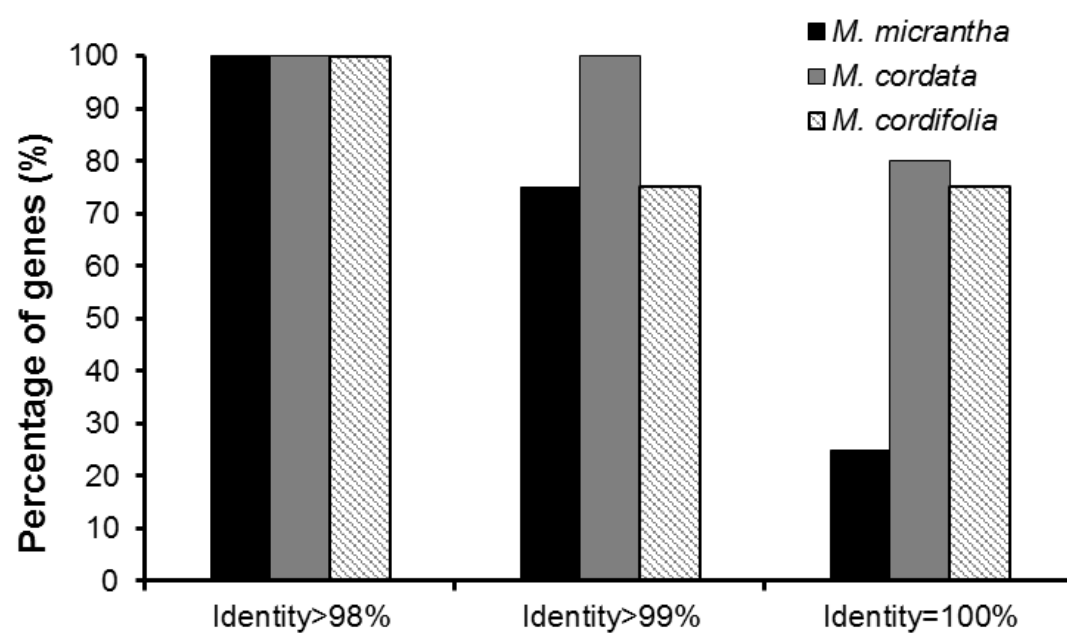

Supplement: Supplementary file 3 — Assessment of per-base sequence accuracy of M. micrantha, M. cordata, and M. cordifolia unigenes. (a) Mapping depth distribution of assembled unigene sequences. Histogram shows the percentages of bases with certain ranges of coverage depth. (b) Identity distribution between assembled unigenes and their corresponding sequences from public databases. (PDF 132 kb) [file 12864_2018_4784_MOESM3_ESM.pdf]

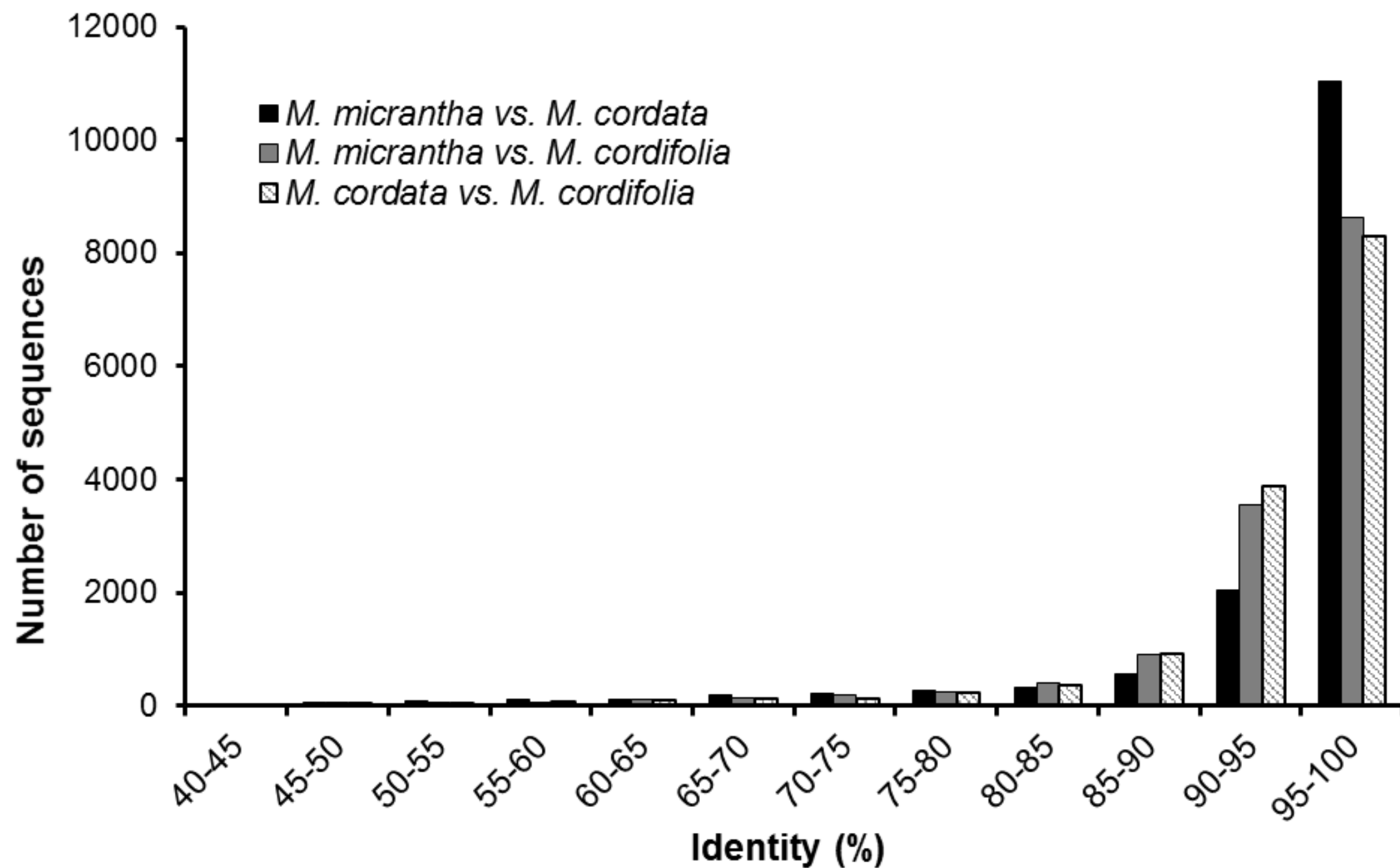

Supplement: Supplementary file 6 — Sequence comparison of M. micrantha, M. cordata, and M. cordifolia ortholog pairs. (PDF 35 kb) [file 12864_2018_4784_MOESM6_ESM.pdf]

a

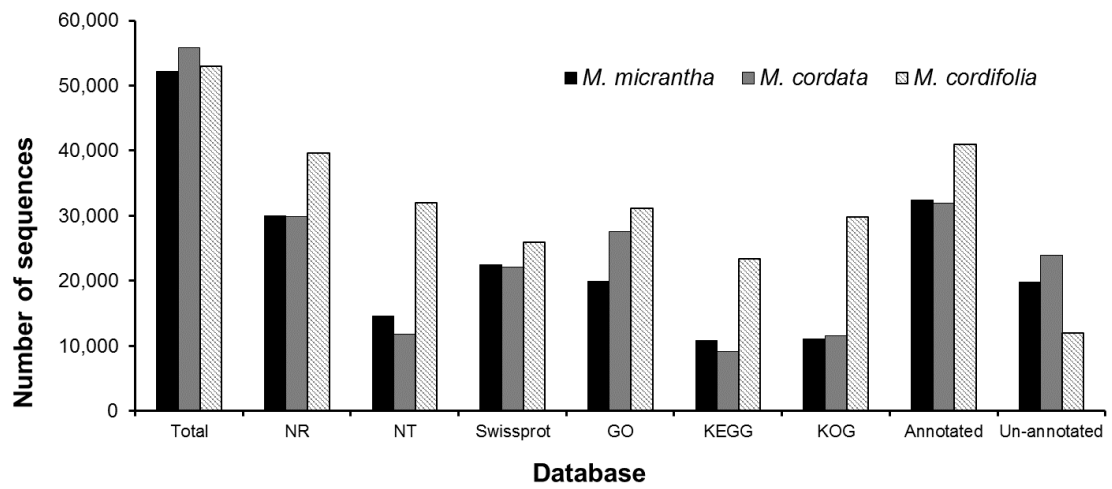

b

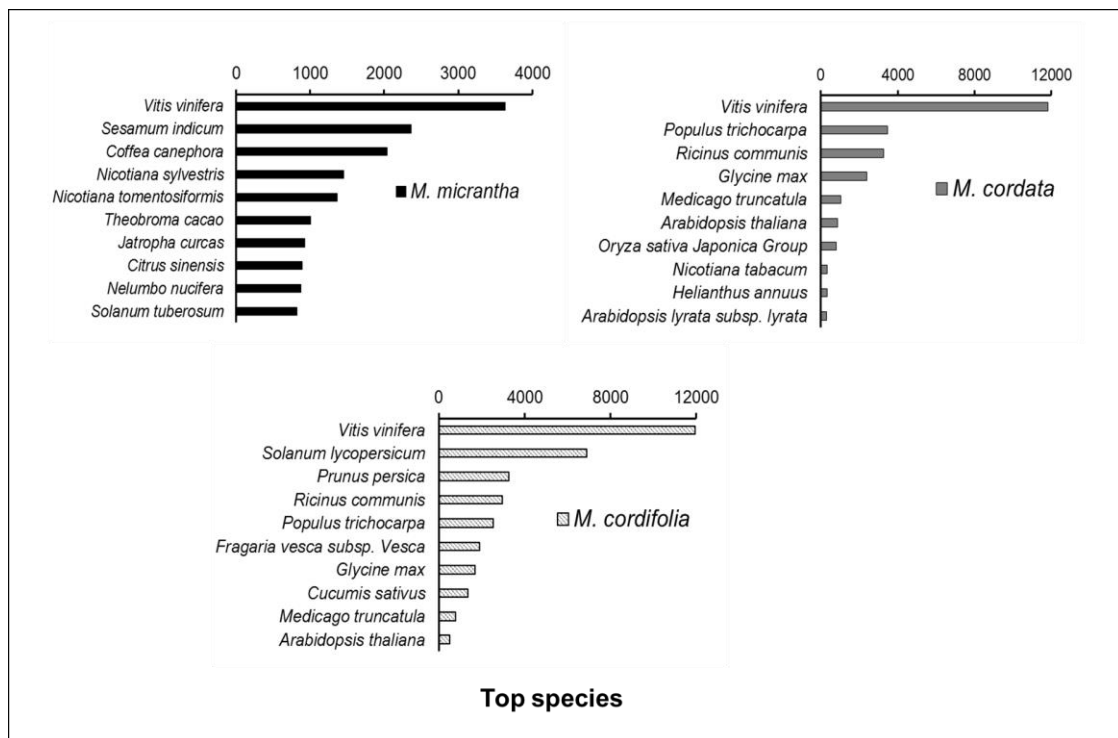

c

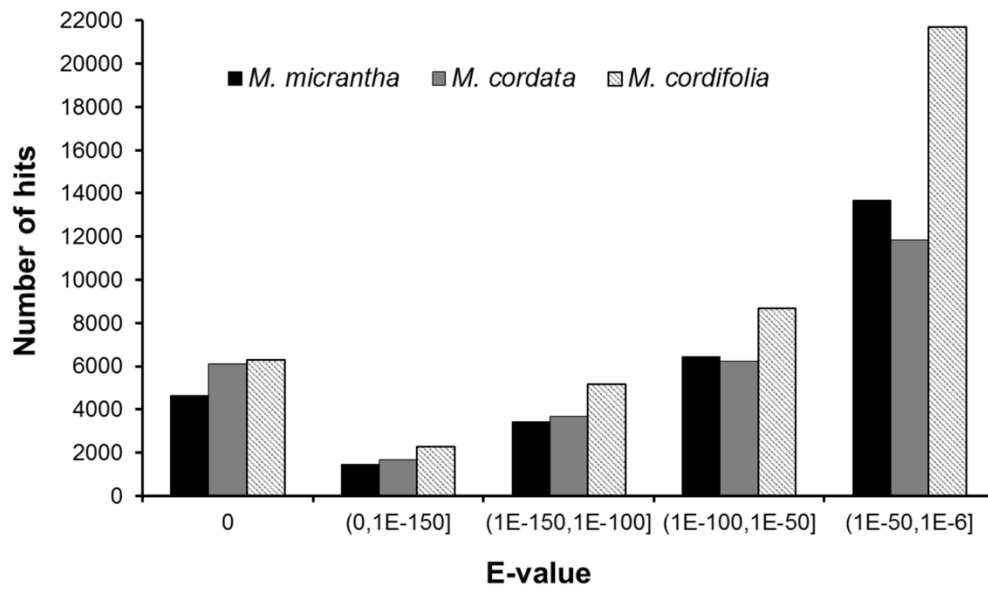

d

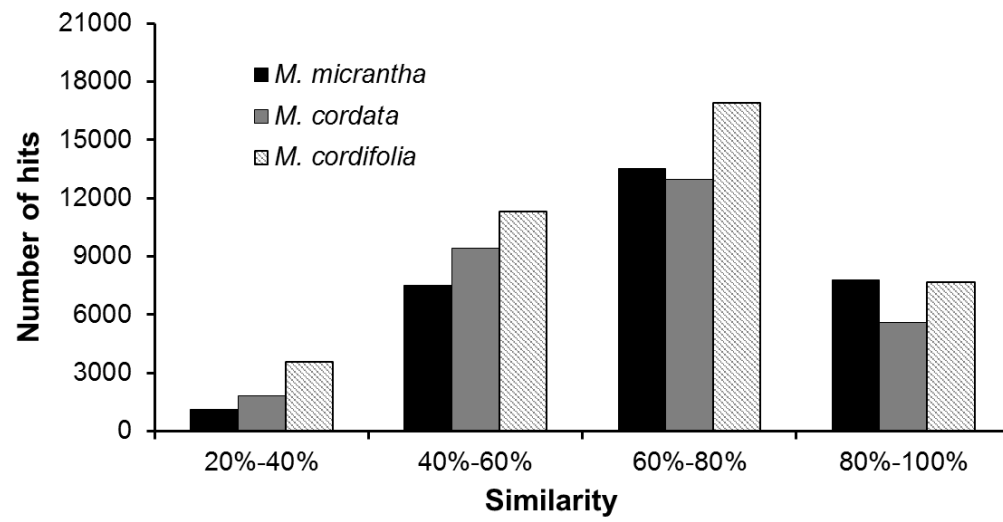

Supplement: Supplementary file 7 — Similarity search and annotation of M. micrantha, M. cordata, and M. cordifolia unigenes. (a) Histogram plot of the data distribution based on search against multiple public databases. (b/c/d) Species/E-value/similarity distributions of top hits for the unigenes based on BLAST search against NCBI non-redundant protein (NR) database. (PDF 414 kb) [file 12864_2018_4784_MOESM7_ESM.pdf]

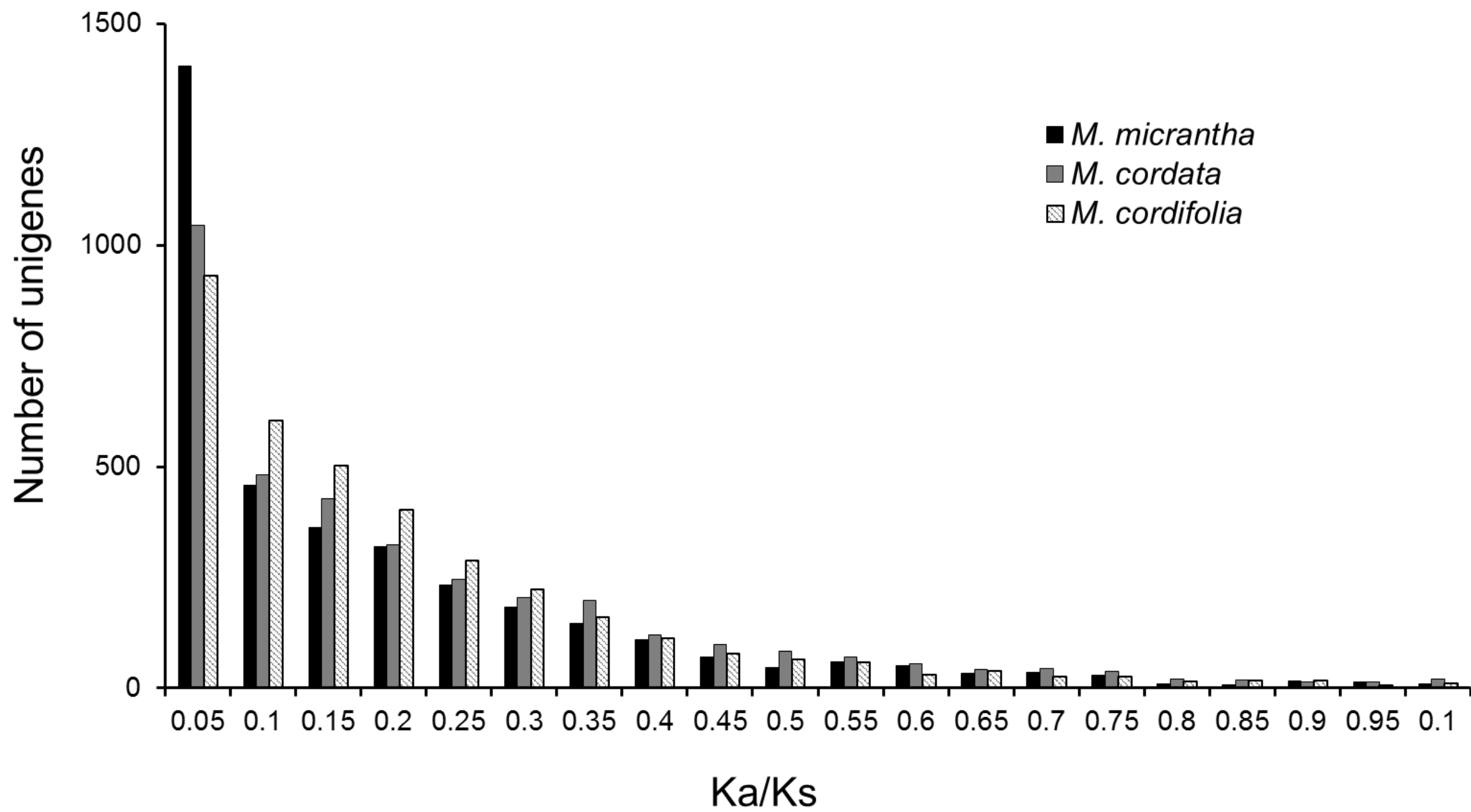

Supplement: Supplementary file 16 — Distribution of branch-specific Ka/Ks for M. micrantha, M. cordata, and M. cordifolia. (PDF 41 kb) [file 12864_2018_4784_MOESM16_ESM.pdf]
