# Supplementary material for: Comparative transcriptome analysis of the invasive weed Mikania micrantha with its native congeners provides insights into genetic basis underlying successful invasion
Source: BMC Genomics. 2018 May 24;19:392. doi: 10.1186/s12864-018-4784-9 (PMC5968712; doi:10.1186/s12864-018-4784-9)
Supplement: Supplementary file 4 — Downloaded nucleotide sequences from public databases and their alignment statistics. (PDF 117 kb) [file 12864_2018_4784_MOESM4_ESM.pdf]

Unigene ID	Unigene length (bp)	Downloaded gene ID	Downloaded gene length (bp)	Match length (bp)	Identity (%)
Mikania micrantha					
mmt|c34589_g1	941	KR262890.1	867	855	100.00
mmt|c51902_g1	214	FJ767894.1	1898	214	100.00
mmt|c12161_g1	3644	KY968832.1	838	372	100.00
mmt|c20581_g3	2324	KJ594363.1	553	553	100.00
mmt|c57017_g1	915	AY270024.1	436	436	100.00
mmt|c20792_g1	2236	KU245489.1	776	776	99.87
mmt|c20792_g3	6763	KJ593955.1	746	746	99.87
mmt|c10205_g2	1020	FJ872512.1	698	665	99.85
mmt|c38247_g1	453	FJ872511.1	1185	418	99.76
mmt|c15192_g1	1380	FJ872513.1	1308	1248	99.76
mmt|c16139_g1	966	KF516995.1	816	807	99.75
mmt|c23065_g1	900	KR262891.1	1041	900	99.56
mmt|c19242_g2	1401	KU245419.1	422	422	99.53
mmt|c25086_g1	388	FJ869889.1	1299	388	99.48
mmt|c59516_g1	332	EU716625.1	1295	332	99.40
mmt|c27807_g1	1029	EU296448.1	1072	1029	98.15
mmt|c7960_g1	1226	EU296447.1	1329	758	98.15
mmt|c13656_g1	460	EU716626.1	1363	460	98.91
mmt|c55428_g1	1096	KR108278.1	960	608	98.85
mmt|c15145_g1	2622	KR011957.1	1567	1185	98.23
M. cordata					
mct|c5106_g1	4226	KX525924.1	713	713	100.00
mct|c35849_g1	3845	AY270025.1	436	436	100.00
mct|c35849_g1	3845	AY270023.1	345	345	100.00
mct|c18784_g1	6688	AF540013.1	652	651	100.00

mct|c24376_g1	4212	KX526973.1	1204	1127	99.91
M. cordifolia					
mco|Unigene25892_MCAF	646	KJ772943.1	651	434	100.00
mco|Unigene6765_MCAF	1418	KJ773684.1	1323	1113	100.00
mco|CL3961.Contig1_MCAF	4808	JQ692098.1	649	649	100.00
mco|CL3961.Contig1_MCAF	4808	KU245413.1	422	422	98.34
