# Supplementary material for: Comparative transcriptome analysis of the invasive weed Mikania micrantha with its native congeners provides insights into genetic basis underlying successful invasion
Source: BMC Genomics. 2018 May 24;19:392. doi: 10.1186/s12864-018-4784-9 (PMC5968712; doi:10.1186/s12864-018-4784-9)
Supplement: Supplementary file 5 — Sanger sequencing-derived sequences and their alignment statistics. (PDF 179 kb) [file 12864_2018_4784_MOESM5_ESM.pdf]

Unigene ID	Primer sequence	Amplified length (bp)	Match length (bp)	Identity (%)
Mikania micrantha				
mmt|c10981_g1	F: 5’ ACCCTTCGACATCAACAG 3’<br>R: 5’ ATTCACCCAAGCCATCAA 3’	589	589	100
mmt|c14245_g1	F: 5’ AATTGTAACTGCAAGAGC 3’<br>R: 5’ GTGAACATTCCACTACCC 3’	705	343	100
mmt|c5343_g1	F: 5’ ATACTTAGGACCCGACCC 3’<br>R: 5’ TCCGTCATGAACTCACTC 3’	525	525	100
mmt|c17105_g1	F: 5’ ACTCTGTCCACCTTGCTT 3’<br>R: 5’ GTGTTTATGCGTTTCCAT 3’	645	478	100
mmt|c10109_g1	F: 5’ AACCACCCTCGATGAATA 3’<br>R: 5’ AATGCGGGCAGCCTTTAC 3’	482	70	100
mmt|c10055_g1	F: 5’ F: TTATGAAGCCAAACAGAG 3’<br>R: 5’ TAGACATCGAGTCCGAGA 3’	749	167	100
mmt|c10179_g1	F: 5’ AATTCCCGTTTGCTTCAC 3’<br>R: 5’ TCTTGGATTACCGCCTGA 3’	743	743	100
mmt|c10238_g1	F: 5’ CTCTGAAATGGACCTACA 3’<br>R: 5’ TTCTCATGCTTACGTGGT 3’	566	566	100
mmt|c10243_g1	F: 5’ AGACTTGAAGCTGGAGAA 3’<br>R: 5’ TTGATGCCACATTGAAAC 3’	904	137	100
M. cordata				
mct|c15905_g1	F: 5’ ACCCTTCGACATCAACAG 3’<br>R: 5’ ATTCACCCAAGCCATCAA 3’	589	589	100
mct|c25241_g1	F: 5’ AATTGTAACTGCAAGAGC 3’<br>R: 5’ GTGAACATTCCACTACCC 3’	722	343	100
mct|c2917_g1	F: 5’ ATACTTAGGACCCGACCC 3’<br>R: 5’ TCCGTCATGAACTCACTC 3’	525	525	100
mct|c26246_g1	F: 5’ ACTCTGTCCACCTTGCTT 3’	652	485	100

	R: 5’ GTGTTTATGCGTTTCCAT 3’			
mct|c22972_g1	F: 5’ AACCACCCTCGATGAATA 3’<br>R: 5’ AATGCGGGCAGCCTTTAC 3’	860	109	100
mct|c24514_g1	F: 5’ F: TTATGAAGCCAAACAGAG 3’<br>R: 5’ TAGACATCGAGTCCGAGA 3’	761	166	100
mct|c6882_g1	F: 5’ AATTCCCGTTTGCTTCAC 3’<br>R: 5’ TCTTGGATTACCGCCTGA 3’	743	743	100
mct|c27268_g2	F: 5’ CTCTGAAATGGACCTACA 3’<br>R: 5’ TTCTCATGCTTACGTGGT 3’	566	566	100
mct|c29419_g1	F: 5’ AGACTTGAAGCTGGAGAA 3’<br>R: 5’ TTGATGCCACATTGAAAC 3’	912	146	100
M. cordifolia				
mco|Unigene24741_MCAF	F: 5’ ACCCTTCGACATCAACAG 3’<br>R: 5’ ATTCACCCAAGCCATCAA 3’	589	589	100
mco|CL5035.Contig1_MCAF	F: 5’ AATTGTAACTGCAAGAGC 3’<br>R: 5’ GTGAACATTCCACTACCC 3’	713	343	100
mco|Unigene18995_MCAF	F: 5’ ATACTTAGGACCCGACCC 3’<br>R: 5’ TCCGTCATGAACTCACTC 3’	525	525	100
mco|Unigene23068_MCAF	F: 5’ ACTCTGTCCACCTTGCTT 3’<br>R: 5’ GTGTTTATGCGTTTCCAT 3’	658	485	100
mco|CL7442.Contig1_MCAF	F: 5’ AACCACCCTCGATGAATA 3’<br>R: 5’ AATGCGGGCAGCCTTTAC 3’	873	109	100
mco|CL6138.Contig1_MCAF	F: 5’ F: TTATGAAGCCAAACAGAG 3’<br>R: 5’ TAGACATCGAGTCCGAGA 3’	734	167	100
mco|Unigene7186_MCAF	F: 5’ AATTCCCGTTTGCTTCAC 3’<br>R: 5’ TCTTGGATTACCGCCTGA 3’	743	743	100
mco|CL3569.Contig1_MCAF	F: 5’ CTCTGAAATGGACCTACA 3’<br>R: 5’ TTCTCATGCTTACGTGGT 3’	566	566	100
mco|CL2809.Contig1_MCAF	F: 5’ AGACTTGAAGCTGGAGAA 3’	896	142	100

	R: 5’ TTGATGCCACATTGAAAC 3’
