# Supplementary material for: Comparative transcriptome analysis of the invasive weed Mikania micrantha with its native congeners provides insights into genetic basis underlying successful invasion
Source: BMC Genomics. 2018 May 24;19:392. doi: 10.1186/s12864-018-4784-9 (PMC5968712; doi:10.1186/s12864-018-4784-9)
Supplement: Supplementary file 10 — KOG classification for M. micrantha, M. cordata, and M. cordifolia unigenes. A total of 11,127, 11,497, and 29,854 unigenes, respectively, were grouped into 26 clusters of ortholog group terms. (PDF 74 kb) [file 12864_2018_4784_MOESM10_ESM.pdf]

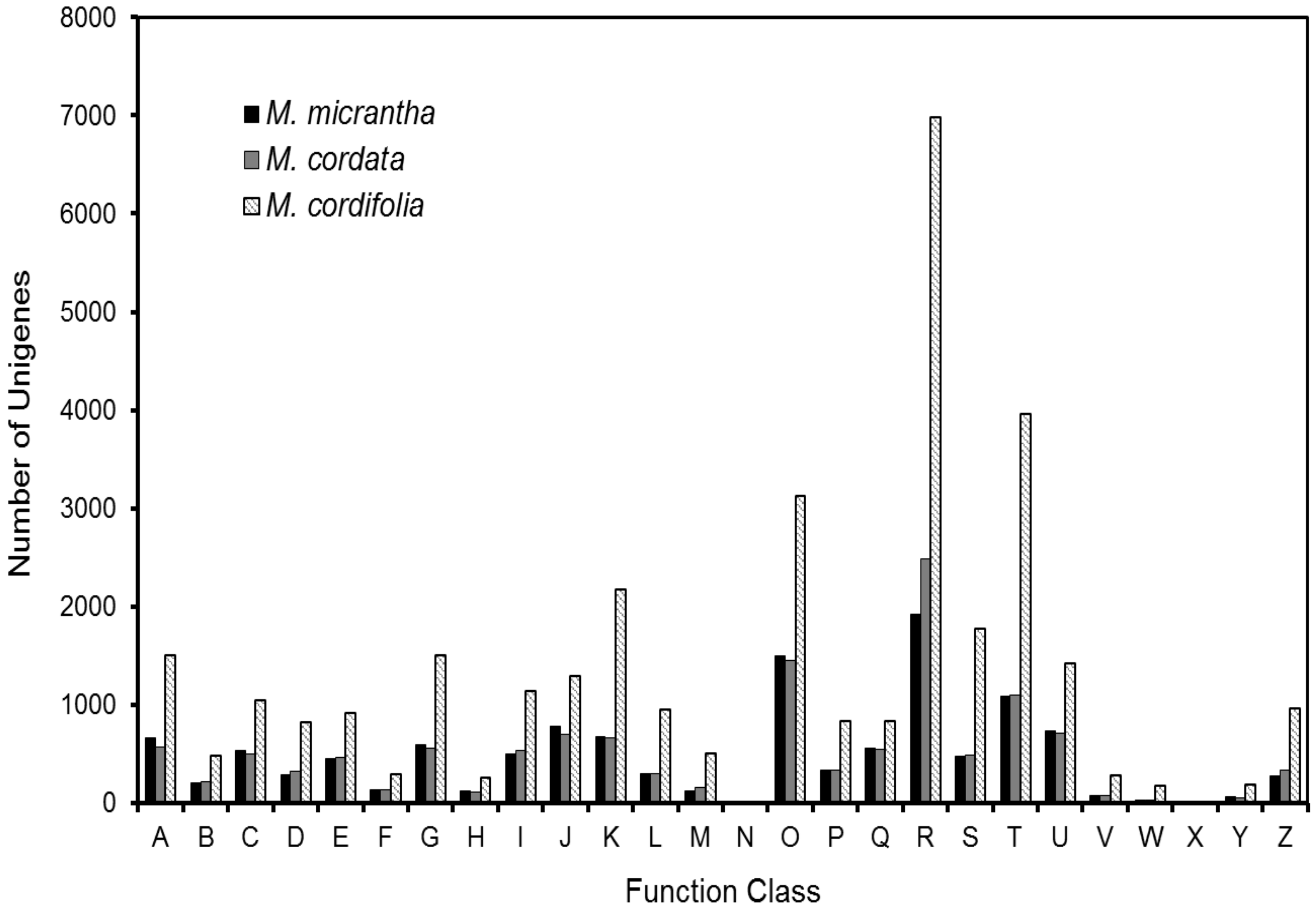

A: RNA processing and modification
B: Chromatin structure and dynamics
C: Energy production and conversion
D: Cell cycle control, cell division, chromosome partitioning
E: Amino acid transport and metabolism
F: Nucleotide transport and metabolism
G: Carbohydrate transport and metabolism
H: Coenzyme transport and metabolism
I: Lipid transport and metabolism
J: Translation, ribosomal structure and biogenesis
K: Transcription
L: Replication, recombination and repair
M: Cell wall/membrane/envelope biogenesis
N: Cell motility
O: Posttranslational modification, protein turnover, chaperones
P: Inorganic ion transport and metabolism
Q: Secondary metabolites biosynthesis, transport and catabolism
R: General function prediction only
S: Function unknown
T: Signal transduction mechanisms
U: Intracellular trafficking, secretion, and vesicular transport
V: Defense mechanisms
W: Extracellular structures
X: Unamed protein
Y: Nuclear structure
Z: Cytoskeleton
