# Supplementary material for: Comparative transcriptome analysis of the invasive weed Mikania micrantha with its native congeners provides insights into genetic basis underlying successful invasion
Source: BMC Genomics. 2018 May 24;19:392. doi: 10.1186/s12864-018-4784-9 (PMC5968712; doi:10.1186/s12864-018-4784-9)
Supplement: Supplementary file 11 — Number of differentially expressed genes (DEGs) under different dispersion values. (PDF 49 kb) [file 12864_2018_4784_MOESM11_ESM.pdf]

Dispersion	#DEGs in *M. micrantha-M. cordata* (up-/down-regulated)	#DEGs in *M. micrantha-M. cordifolia* (up-/down-regulated)	#DEGs shared (up-/down-regulated)
0.05	2948 (1918/1030)	2990 (2155/835)	1300 (950/350)
0.1	2954 (1922/1032)	2994 (2159/835)	1308 (958/350)
0.15	2954 (1922/1032)	2994 (2159/835)	1308 (958/350)
0.2	2088 (1337/751)	2344 (1724/620)	891 (650/241)

Table 2. List of genes that were up- and down-regulated in M. micrantha compared with both M. cordata and M. cordifolia

Gene name	Counts in M. micrantha	Counts in M. cordata	logFC	logCPM	P-Value	FDR
c32279_g1_i3	1914	87	6.299	7.526	9.17E-08	0.001
c26998_g2_i1	297	10	6.708	4.826	1.21E-07	0.001
c34356_g2_i1	217	12	5.997	4.380	9.95E-07	0.005
c35366_g6_i1	331	25	5.559	5.001	2.17E-06	0.007
c34106_g1_i1	266	20	5.563	4.683	2.26E-06	0.007
c24371_g1_i1	144	10	5.664	3.789	3.95E-06	0.011
c35531_g4_i1	339	34	5.153	5.045	5.66E-06	0.014
c34509_g2_i3	144	12	5.405	3.794	7.04E-06	0.014
c33340_g1_i1	462	51	5.017	5.498	7.64E-06	0.014
c34596_g2_i4	174	17	5.183	4.076	7.68E-06	0.014
c34856_g1_i3	269	29	5.047	4.713	1.03E-05	0.017
c29976_g1_i2	128	13	5.122	3.630	1.24E-05	0.018
c30994_g5_i1	196	22	4.986	4.255	1.38E-05	0.019
c9815_g1_i1	107	10	5.235	3.365	1.64E-05	0.019
c29997_g1_i1	225	27	4.892	4.459	1.81E-05	0.019
c9571_g1_i1	145	17	4.920	3.819	1.82E-05	0.019
c30994_g3_i1	357	47	4.763	5.133	1.84E-05	0.019
c34710_g2_i2	168	20	4.900	4.034	2.00E-05	0.019
c34710_g1_i1	102	10	5.166	3.297	2.03E-05	0.019
c27250_g1_i2	424	59	4.684	5.385	2.10E-05	0.019
c24371_g2_i1	140	16	4.955	3.766	2.12E-05	0.019
c32972_g11_i1	111	12	5.030	3.425	2.41E-05	0.020
c28111_g1_i2	573	85	4.593	5.824	2.70E-05	0.022
c34004_g1_i1	448	65	4.624	5.467	2.78E-05	0.022
c9571_g2_i1	114	15	4.751	3.474	3.50E-05	0.026
c34345_g1_i1	222	35	4.501	4.454	5.19E-05	0.037
c34073_g4_i1	134	20	4.574	3.717	5.67E-05	0.039
c32961_g1_i1	139	23	4.427	3.777	6.82E-05	0.044
c10622_g2_i1	19	3807	-5.799	6.691	6.61E-07	0.004
c37112_g1_i1	30	2172	-4.332	5.930	6.88E-05	0.044
c35128_g1_i2	30	2150	-4.318	5.916	7.22E-05	0.045
